# The impact of vaccines for diarrhoea on antibiotic use among children in five low-resource settings: a comparative simulation study

**DOI:** 10.1016/S2214-109X(24)00378-4

**Published:** 2024-11-20

**Authors:** Elizabeth T Rogawski McQuade, Stephanie A Brennhofer, Sarah E Elwood, Joseph A Lewnard, Jie Liu, Eric R Houpt, James A Platts-Mills

**Affiliations:** a Department of Epidemiology, Rollins School of Public Health, Emory University, Atlanta, GA, USA; b Division of Infectious Diseases & International Health, University of Virginia, Charlottesville, VA, USA; c Division of Epidemiology, School of Public Health, University of California, Berkeley, CA, USA; d School of Public Health, Qingdao University, Qingdao, Shandong, China

## Abstract

**Background:**

Vaccines for diarrhoea could have the ancillary benefit of preventing antibiotic use. We aimed to quantify and compare the expected impact of enteric vaccines on antibiotic use via Monte Carlo simulations.

**Methods:**

We analysed data from a longitudinal birth cohort, which enrolled children from 2009 to 2012 from Bangladesh, India, Nepal, Pakistan, and Tanzania. We used Monte Carlo simulations to estimate hypothetical vaccine impact in nine vaccination scenarios (including six single vaccines and three combination vaccines) on antibiotic- treated diarrhoea, overall antibiotic courses, and antibiotic exposures to bystander pathogens. For each vaccine scenario, we randomly selected target pathogen-specific diarrhoea episodes to be prevented according to the specified vaccine efficacy and estimated the absolute and relative differences in incidence of antibiotic use outcomes between vaccine and no vaccine scenarios.

**Findings:**

Among 1119 children, there were 3029 (135·3 courses per 100 child-years) antibiotic-treated diarrhoea episodes. Based on simulated results, a *Shigella* vaccine would cause the greatest reductions compared with the other single pathogen vaccines in antibiotic courses for all-cause diarrhoea (6·1% relative reduction; –8·2 courses per 100 child-years [95% CI –9·4 to –7·2]), antibiotic courses overall (1·0% relative reduction; –8·2 courses per 100 child-years [–9·4 to –7·2]), and antibiotic exposures to bystander pathogens (1·2% relative reduction; –15·9 courses per 100 child-years [–18·5 to –13·8]). An adenovirus–norovirus–rotavirus vaccine would cause the greatest reductions in antibiotic use (12·2 courses per 100 child-years [–13·7 to –11·0]) compared with the other combination vaccines. However, projected vaccine effects on antibiotic use in 2021 were 45–74% smaller than those estimated in 2009–12 accounting for reductions in diarrhoea incidence in the past decade.

**Interpretation:**

Vaccines for enteric pathogens could result in up to 8–12 prevented courses of antibiotics per 100 vaccinated children per year. Combination vaccines will probably be necessary to achieve greater than 1% reductions in total antibiotic use among children in similar low-resource settings.

**Funding:**

Wellcome Trust and Bill & Melinda Gates Foundation.

## Introduction

In low-resource settings, children **y**ounger than 5 years have approximately two diarrhoea episodes per year on average.^1^ Contrary to WHO guidelines stipulating that only dysentery should be treated with antibiotics,^2^ many non-dysenteric diarrhoea episodes are treated empirically.^3^ Because enteric viruses are a major cause of diarrhoea,^4^, ^5^ antibiotic treatment is often ineffective and unnecessarily leads to antibiotic selection pressure on bystander bacterial pathogens carried asymptomatically at the time of treatment.^6^ Given the high burden of diarrhoea in low-resource settings, antibiotic overuse in young children is a probable contributor to antimicrobial resistance.^6^, ^7^

Antibiotic stewardship interventions aim to improve rational antibiotic use, but demand for antibiotics and treatment behaviour among patients and providers alike is difficult to change, especially in settings where antibiotics are available in the community.^8^ Given the barriers to preventing unnecessary antibiotic use once a child is ill, strategies to prevent the illness from occurring that would eliminate the need for treatment might be more effective in reducing antibiotic use;^9^ this is particularly true for diarrhoea episodes in which antibiotics are indicated and therefore unethical to withhold, such as for shigellosis and campylobacteriosis. Fluoroquinolone and macrolide resistance is of particular concern for these illnesses, which increases the urgency of the need for preventive interventions.^10^ Vaccines offer a primary means of preventing illness, and pneumococcal conjugate vaccines and rotavirus vaccines have documented efficacy in preventing antibiotic use in low-income and middle-income countries (LMICs).^11^

Although rotavirus vaccines are the only vaccines targeting diarrhoea that have been introduced into national immunisation programmes in many countries, there are several enteric vaccines in the pipeline.^12^ An important component of the vaccine value proposition is the magnitude of antibiotic use that these vaccines could prevent.^13^ We previously predicted that *Shigella* vaccines would have a relatively limited effect in isolation on total antibiotic use and antibiotic exposures for bystander enteric pathogens.^14^ It is probable that multiple enteric vaccines, potentially in combination vaccine formulations, will be necessary to make substantial reductions in use and resulting antimicrobial resistance.

**Research in context**

**Evidence before this study**

WHO has emphasised the need to understand the potential reductions in antibiotic use that could be achieved with vaccines. We searched PubMed for articles published in English from Jan 1, 1990 to Aug 21, 2024 (when the search was performed) using the terms: (diarrhoea OR diarrhea) AND (vaccine) AND (antibiotic use) AND (simulation OR prediction). Of the 41 identified publications, two publications estimated reductions in antibiotic use after introduction of a rotavirus or *Shigella* vaccine, but none compared the potential impact of a range of enteric vaccines.

**Added value of this study**

This study expands on previous work on the simulated effects of *Shigella* vaccines by comparing the impact of six individual vaccines and three combination vaccines for diarrhoea on antibiotic use. We used data from a longitudinal birth cohort that followed up with children for their first 2 years of life to quantify the potential impact of introducing different enteropathogen vaccines on antibiotic use outcomes. Across sites, a *Shigella* vaccine would be expected to have the greatest impact among individual vaccines. A combination enteric virus vaccine covering rotavirus, norovirus, and adenovirus 40 and 41 would have the greatest impact among combination vaccines assessed.

**Implications of all the available evidence**

Children in low-resource settings are frequently treated with antibiotics for diarrhoea and vaccines for diarrhoea could have the ancillary benefit of preventing antibiotic use with implications for antimicrobial resistance. Prioritisation of development of vaccines for diarrhoea should consider potential impacts on antibiotic use, and combination vaccines would best achieve goals for limiting antibiotic resistance.

In this simulation study, we compared the expected impact of six individual enteric vaccines and three combination enteric vaccines on antibiotic use for diarrhoea, antibiotic use overall, and bystander antibiotic exposures (ie, exposure to pathogens carried asymptomatically at the time of treatment) in the Etiology, Risk Factors, and Interactions of Enteric Infections and Malnutrition and the Consequences for Child Health and Development (MAL-ED) study. We model impacts for vaccines (under development) against *Shigella,* enterotoxigenic *Escherichia coli* (ETEC), norovirus, *Campylobacter*, rotavirus, and adenovirus. These pathogens are among the leading causes of diarrhoea among children in low-resource settings^4^, ^5^ and are targeted by existing vaccines (rotavirus) or vaccines in the development pipeline. We also evaluated combination viral vaccines (norovirus–rotavirus and adenovirus–norovirus–rotavirus) for pathogens that have similar age distributions of disease and might be co-formulated, and a combination bacterial vaccine (*Shigella*–ETEC), which is in development.^15^ These estimates inform which enteric vaccines should be prioritised for development and future use based on their potential contribution to limiting antibiotic use compared with rotavirus vaccines as an existing benchmark.

## Methods

### Study design and participants

The study design for MAL-ED has been previously detailed.^5^ Children were enrolled at birth (aged <17 days) from 2009 to 2012 and were followed up for 2 years. The five MAL-ED sites (out of a total of eight) that did not have routine rotavirus vaccine use at the time of the study were included in the analyses: Dhaka, Bangladesh; Vellore, India; Bhaktapur, Nepal; Naushero Feroze, Pakistan; and Haydom, Tanzania. Fieldworkers visited the homes of participants twice per week to ascertain any illness and daily antibiotic use, including specific drug classes as reported by the caregiver during the 2-year follow-up. Non-diarrhoeal surveillance stools were collected monthly and during diarrhoea episodes. Diarrhoea episodes were defined as three or more loose stools in 24 h or presence of blood in the stool. The MAL-ED study involved human participants, and all sites received ethical approval. Caregivers provided written informed consent for their child to participate.

### Procedures

The QIAamp Fast DNA Stool Mini Kit (Qiagen, Hilden, Germany) was used to extract total nucleic acid from stool samples.^16^ Enteropathogens were detected via quantitative PCR using AgPath One Step real-time PCR kit (Thermo-Fisher, Waltham, MA, USA).^5^ Adenovirus types 40 and 41 were detected using the fiber gene; norovirus GII using the GII *ORF1–2* gene; rotavirus using the *NSP3* gene; *Campylobacter* spp using the *cadF* (*Campylobacter jejuni* and *Campylobacter coli*) and *cpn60* (*Campylobacter* spp) genes; ETEC using the *elt* and *est* genes encoding LT, STh, and STp; and *Shigella* using the *ipaH* gene.^5^ Positive pathogen detection occurred at a cycle threshold below 35.

We assessed vaccine impact on the following outcomes for all antibiotics and fluoroquinolones and macrolides specifically, which are recommended for the treatment of diarrhoea: antibiotic-treated bacterial diarrhoea episodes, antibiotic-treated viral or parasitic diarrhoea episodes, antibiotic-treated diarrhoea episodes of any cause, overall antibiotic courses, and antibiotic exposures to bystander pathogens. Distinct antibiotic courses were defined if separated by at least 2 days of no antibiotic use.^3^ Antibiotic courses were attributed to diarrhoea episodes if the antibiotic course overlapped with reported diarrhoea symptoms on at least 1 day and did not overlap with an episode of acute lower respiratory infection.^6^ Diarrhoea was attributed to specific causes using the episode-specific attributable fraction (AFe) over 0·5. AFes were calculated as previously as 1 – (1/ORe), where ORe was the pathogen-specific and quantity-specific odds ratio (OR) from a generalised linear mixed-effects logistic regression model.^5^ The outcome of the model was diarrhoeal stool versus non-diarrhoeal stool, the exposure was each pathogen quantity detected, and the model was adjusted for quantities of other pathogens,^5^ sex, age, sample testing batch, and random effects for site and individual. An interaction term between pathogen quantity and age was included, as well as a quadratic term for pathogen quantity if it improved model fit by the Akaike information criterion. Severe diarrhoea was defined as a modified Vesikari score higher than 6 and had a different vaccine efficacy applied compared with non-severe diarrhoea (see below).^5^

We identified bystander exposures by linking each antibiotic course to the most recent stool sample within the previous 30 days.^6^ We considered enteropathogenic bacteria present in at least 5% of non-diarrhoeal stools as bystander pathogens (atypical enteropathogenic *E coli*], *Campylobacter* spp, enteroaggregative *E coli*], ETEC, typical enteropathogenic *E coli*, and *Shigella* spp). If these pathogens were detected in the linked stool, they were considered bystanders to the antibiotic course unless they were identified as the cause of diarrhoea prompting antibiotic treatment.^6^

We considered six two-dose single pathogen vaccines in hypothetical use in the MAL-ED study: *Shigella, Campylobacter,* ETEC, norovirus, rotavirus, and adenovirus, and three combination pathogen vaccines: *Shigella*–ETEC, norovirus–rotavirus, and adenovirus–norovirus–rotavirus as nine vaccination scenarios. The viral vaccines (adenovirus, norovirus, and rotavirus) were simulated to be administered at ages 2 months and 4 months, and the bacterial vaccines (*Campylobacter*, ETEC, and *Shigella*) were administered at ages 6 months and 9 months (appendix p 4). Rotarix, one of the two rotavirus vaccines available to date, is given globally in two doses at age 2 months and 4 months.^17^ Because there are no guidelines for a norovirus or adenovirus vaccine, they were given the same vaccine schedule as rotavirus to keep dosing schedules comparable. The dosing schedule for the bacterial vaccines was based on WHO's Preferred Product Characteristics for vaccines against ETEC and *Shigella,* which recommend complete immunisation against ETEC by age 9 months and *Shigella* by age 12 months.^18^, ^19^ For consistency, *Campylobacter* was given the same dosing schedule as *Shigella* and ETEC in the absence of guidelines. Vaccine scenarios assumed 100% vaccine coverage. We performed a sensitivity analysis where vaccine coverage was 60%, which approximates observed rotavirus vaccine uptake in LMICs.^20^

As previously,^14^ we simulated vaccine efficacy (ie, proportion of episodes that are prevented by the vaccine) using a beta distribution (α=6, β=4) to target a mean efficacy of 60% against severe disease 14 days after administration of the second dose, and such that 80% of all simulated values were within an absolute 20% above and below the mean (appendix p 4). To achieve a lower mean efficacy for non-severe disease, the simulated efficacies for severe disease were multiplied by two-thirds, to yield a mean efficacy of 40%.

Assumed vaccine efficacy against severe disease was also based on WHO's Preferred Product Characteristics for *Shigella* and ETEC. Lower vaccine efficacy for non-severe compared with severe disease was observed in rotavirus vaccine trials.^21^ Reduced efficacy for non-severe disease is also expected for other enteric pathogens, as was observed in a controlled human infection model study of a *Shigella* vaccine.^22^ Vaccine viral replication and immunity stimulation is expected 1–2 weeks post vaccination.^23^ For this reason, the follow-up period in pivotal rotavirus vaccine trials started 2 weeks after the final dose.^24^ Therefore, we assumed full vaccine efficacy 14 days after the second dose and half of the full vaccine efficacy was assumed 14 days after the first dose.

### Statistical analysis

We estimated the incidence of antibiotic use outcomes under each vaccine scenario via Monte Carlo simulations with random sampling and replacement to a sample size of 50 000. To calculate the incidence of each outcome, diarrhoea episodes were randomly selected to be prevented at a probability equal to the simulated vaccine efficacy described previously. We compared results with a counterfactual scenario in which no episodes were prevented by vaccination. To extrapolate to diarrhoea episodes that did not have a stool sample collected or were not tested in our dataset, we multiplied the incidence estimates by the proportion:

$$\frac{\mathrm{All diarrhoea episodes in resampled dataset}}{\mathrm{Diarrhoea episodes validly tested by qPCR in resampled dataset}}\left(1\cdot 377\left[95\%CI1\cdot 336-1\cdot 423\right]\right)$$

Furthermore, we multiplied the bystander exposure incidence by the proportion:

$$\frac{\mathrm{All antibiotic courses in resampled dataset}}{\mathrm{Antibiotic courses that could be linked in resampled dataset}}\left(1\cdot 155\left[95\%\text{CI}1\cdot 146-1\cdot 165\right]\right)$$

to extrapolate to the antibiotic courses that could not be linked to a stool in the previous 30 days. Confidence intervals were defined as the 2·5 and 97·5 percentiles generated across 1000 iterations of this procedure, and recalculation of the above multipliers within each iteration ensured that their variability was reflected in the final estimates.

To quantify the reduction in antibiotic use expected with a vaccine, we calculated absolute differences as incidence rate differences:

$$\left({\mathrm{Incidence}}_{\mathrm{vaccine scenario}}-{\mathrm{Incidence}}_{\mathrm{no vaccine scenario}}\right)$$

relative differences as incidence rate ratios:

$$\left(\frac{{\mathrm{Incidence}}_{\mathrm{vaccine scenario}}}{{\mathrm{Incidence}}_{\mathrm{no vaccine scenario}}}\right)$$

and the percent reduction between each vaccine and the no vaccine scenarios:

$$\left(\frac{{\mathrm{Incidence}}_{\mathrm{no vaccine scenario}}{-\mathrm{Incidence}}_{\mathrm{vaccine scenario}}}{{\mathrm{Incidence}}_{\mathrm{no vaccine scenario}}}\right)$$

Analyses were conducted overall and separately for each site. To account for changes in diarrhoea incidence since the MAL-ED study, we conducted a sensitivity analysis in which the incidence of antibiotic-treated diarrhoea in the no vaccine scenario was reduced in proportion to the corresponding country-specific reduction in all-cause diarrhoea incidence between 2013 and 2021, as estimated by the Global Burden of Disease Study from the Institute for Health Metrics and Evaluation (appendix pp 5–7).^25^ We also performed a sensitivity analysis in which diarrhoea cause was assigned based on any pathogen detection (cycle threshold <35) instead of AFe higher than 0·5.

All statistical analyses were performed via R software, version 4.0.2. The statistical analysis code is available online.

### Role of the funding source

The funders of the study had no role in study design, data collection, data analysis, data interpretation, or writing of the report.

## Results

Among 1119 children enrolled in 2009–12, caregivers reported 577·2 antibiotic courses per 100 child-years (n=12 918), of which 116·7 courses per 100 child-years were fluoroquinolones or macrolides (n=2611; table). There were 3029 (135·3 courses per 100 child-years) antibiotic-treated diarrhoea episodes, of which 546 (33·6 courses per 100 child-years) were of bacterial cause and 763 (47·0 courses per 100 child-years) were of viral or parasitic cause—the rest (>50%) were of unknown causes. Across all bystander pathogens, there were 942·6 exposures to antibiotics per 100 child-years (n=18 259).

**Table:** Antibiotic use and bystander pathogen exposures to antibiotics among 1119 children from five sites enrolled in the MAL-ED birth cohort

	**Any antibiotic use**	**Fluoroquinolone or macrolide use**
Antibiotic courses for bacterial diarrhoea episodes*	546 (33·6)	294 (18·1)
Antibiotic courses for parasitic or viral diarrhoea episodes*	763 (47·0)	400 (24·6)
Antibiotic courses for diarrhoea episodes of any cause	3029 (135·3)	944 (42·2)
Antibiotic courses for *Campylobacter jejuni* or *Campylobacter coli* diarrhoea episodes*	7 (0·4)	1 (0·1)
Antibiotic courses for ETEC diarrhoea episodes*	192 (11·8)	112 (6·9)
Antibiotic courses for norovirus diarrhoea episodes*	66 (4·1)	24 (1·5)
Antibiotic courses for rotavirus diarrhoea episodes*	237 (14·6)	135 (8·3)
Antibiotic courses for *Shigella* diarrhoea episodes*	338 (20·8)	196 (12·1)
Antibiotic courses, overall	12 918 (577·2)	2611 (116·7)
Antibiotic exposures to bystander pathogens, overall†	18 259 (942·6)	4319 (223·0)

Data shown are n (rate). Rate is per 100 child-years and extrapolated to all exposures. ETEC=enterotoxigenic *Escherichia coli*.

* Counted among infections, episodes, and exposures in which stools were collected with valid quantitative PCR test results. Rates are extrapolated to all infections, episodes, and exposures.

† Counted among episodes in which a stool was collected in the previous 30 days.

**Figure 1:**
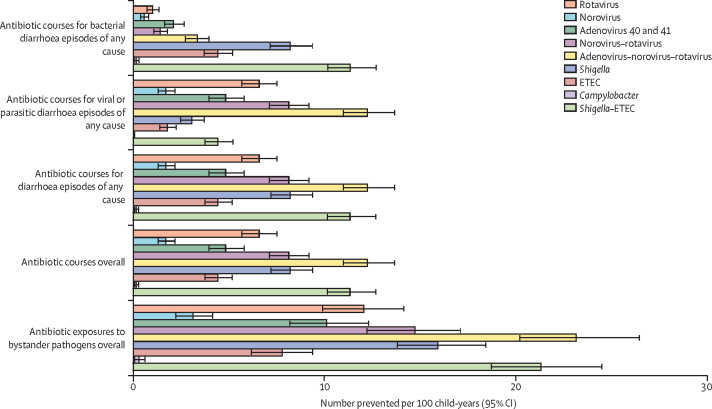
Absolute (incidence rate) differences in antibiotic use outcomes for any drug class for nine enteric pathogen vaccine scenarios compared to the no vaccine scenario with 60% full vaccine efficacies against severe cause-specific diarrhoea at five sites in the MAL-ED birth cohort study ETEC=enterotoxigenic *Escherichia coli*.

**Figure 2:**
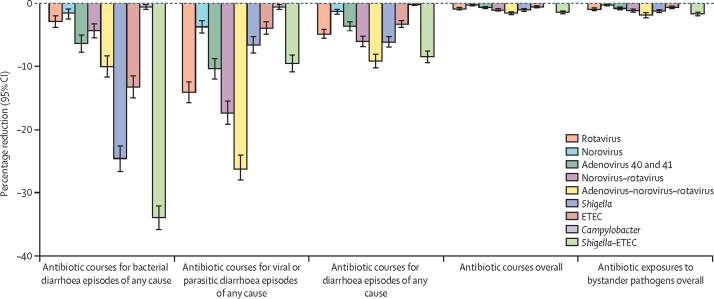
Percentage reductions in antibiotic use of any drug class for nine enteric pathogen vaccine scenarios compared with the no vaccine scenario with 60% full vaccine efficacies against severe cause-specific diarrhoea at five sites in the MAL-ED birth cohort study ETEC=enterotoxigenic *Escherichia coli*.

Under the assumption of 100% vaccination coverage, a *Shigella* vaccine would be expected to cause the greatest reduction compared with the other five single pathogen vaccines in antibiotic courses for bacterial diarrhoea episodes (relative 24·5% reduction; –8·2 courses per 100 child-years [95% CI –9·4 to –7·2]), antibiotic courses for diarrhoea episodes of any cause (relative 6·1% reduction; –8·2 courses per 100 child-years [–9·4 to –7·2]), antibiotic courses overall (relative 1·0% reduction; –8·2 courses per 100 child-years [–9·4 to –7·2]), and antibiotic exposures to bystander pathogens overall (relative 1·2% reduction; –15·9 courses per 100 child-years [–18·5 to –13·8]; Figure 1, Figure 2; appendix pp 8–9). A rotavirus vaccine would be expected to cause the greatest relative reduction (14·1%) in antibiotic courses for viral or parasitic diarrhoea episodes with 6·6 courses (95% CI 5·7–7·5) per 100 child-years prevented (Figure 1, Figure 2; appendix pp 8–9). Conversely, a *Campylobacter* vaccine would have minimal impact (<1% relative reduction; <0·5 courses or courses per 100 child-years; Figure 1, Figure 2; appendix pp 8–9). The absolute reductions of all vaccines with 100% coverage were approximately 2–3 times greater among all antibiotics compared with fluoroquinolones and macrolides (appendix pp 10–12). However, relative reductions were about 1·5–3·0 times greater among fluoroquinolones and macrolides compared with all antibiotics (appendix pp 10–11, 13).

An adenovirus–norovirus–rotavirus combination vaccine given at ages 2 months and 4 months would be expected to cause the greatest relative reductions compared with the other three combination vaccines in antibiotic courses for viral or parasitic diarrhoea episodes (relative 26·1% reduction; –12·2 courses per 100 child-years [95% CI –13·7 to –11·0]), antibiotic courses for diarrhoea episodes of any cause (relative 9·1% reduction; –12·2 courses per 100 child-years [–13·7 to –11·0]), antibiotic courses overall (relative 1·5% reduction; –12·2 courses per 100 child-years [–13·7 to –11·0]), and antibiotic exposures to bystander pathogens overall (relative 1·8% reduction; –23·1 courses per 100 child-years [–26·5 to –20·2]; Figure 1, Figure 2; appendix pp 8–9). A *Shigella*–ETEC combination vaccine would have a substantial reduction in antibiotic courses for bacterial diarrhoea episodes (relative 33·8% reduction; –11·3 courses per 100 child-years [–12·7 to –10·2]).

**Figure 3:**
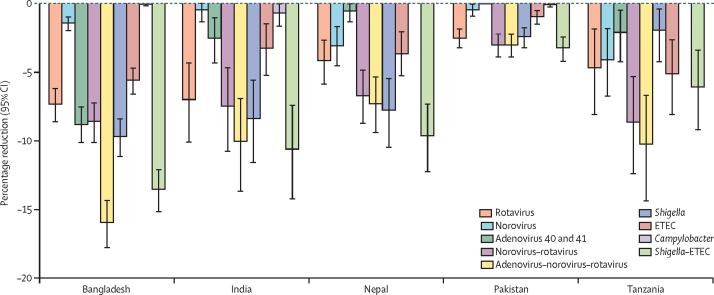
Percentage reductions in antibiotic courses for diarrhoea episodes of any cause (any drug class) for nine enteric pathogen vaccine scenarios compared with the no vaccine scenario with 60% full vaccine efficacies against severe cause-specific diarrhoea at five sites in the MAL-ED birth cohort study ETEC=enterotoxigenic *Escherichia coli*.

The vaccines expected to prevent the most antibiotic courses varied by site (appendix pp 14–16). Among single vaccines, a Shigella vaccine would have the biggest impact in Bangladesh, India, and Nepal, a rotavirus vaccine would have the biggest impact in Pakistan, and an ETEC vaccine would have the biggest impact in Tanzania. Among combination vaccines, the adenovirus–norovirus–rotavirus vaccine would have the biggest impact in Bangladesh and Tanzania and the Shigella–ETEC vaccine would have the biggest impact elsewhere. The greatest absolute reductions in antibiotic courses for diarrhoea would be expected in the Bangladesh site. The percentage reduction in antibiotic courses for diarrhoea of any cause ranged from 2·0% to 9·7% across sites for the Shigella vaccine, and from 3·0% to 15·9% across sites for the adenovirus–norovirus–rotavirus vaccine (figure 3; appendix pp 14–15).

In a sensitivity analysis to project vaccine effects on antibiotic use outcomes in 2021, all the considered vaccines caused lower absolute reductions in antibiotic courses for diarrhoea episodes of any cause compared with the primary analysis (appendix pp 17–19). Depending on the country, diarrhoea incidence was estimated to have declined by 45–74% between 2013 (ie, the midpoint of MAL-ED follow-up) and 2021, leading to proportional reductions in the absolute impact of the vaccines on antibiotic use. For example, in Bangladesh a *Shigella* vaccine would be expected to prevent 1·0 (95% CI 0·7–1·2) antibiotic course for diarrhoea per 100 child-years in 2021, compared with 3·8 (3·0–4·5) antibiotic courses for diarrhoea per 100 child-years at the time of the MAL-ED study.

In a second sensitivity analysis, in which diarrhoea cause was defined by a cycle threshold below 35, the incidence of pathogen-specific diarrhoea was higher than that when defined by AFe above 0·5, most strikingly for *C jejuni* and *C coli* (69·1 *vs* 2·3 episodes per 100 child-years; appendix p 20). Correspondingly, the number of prevented outcomes increased for all vaccines, except for the bystander exposure outcomes, since the total number of bystander exposures decreased under the revised definition (appendix pp 21–22). A *Campylobacter* vaccine went from preventing 0·2 (95% CI 0·0–0·3) antibiotic courses for diarrhoea episodes of any cause per 100 child-years to 11·5 courses per 100 child-years (10·0–13·0).

In the third sensitivity analysis, with 60% vaccine coverage, estimates of all outcomes were reduced by an expected 40% (appendix pp 23–26).

## Discussion

Vaccines for enteric pathogens could result in important absolute reductions in antibiotic use for diarrhoea—more than one prevented course per ten vaccinated children per year—with the additional benefit of fewer antibiotic exposures for bystander pathogens. Among individual enteric vaccines, *Shigella* vaccines are expected to make the largest impact on antibiotic use overall. However, the relative reductions in total antibiotic use attributable to individual vaccines are small (0·0–1·0%) given the many causes of antibiotic use in this population.^3^ Combination vaccines will probably be necessary to make observable relative reductions in total antibiotic use among children in low-resource settings, and those reductions might still be modest (<5%). We found that the triple combination viral vaccine would have the greatest impact on antibiotic use overall, followed by the *Shigella*–ETEC combination. However, this ranking was geography-specific and matched the site-level burden of pathogen-specific antibiotic courses for diarrhoea previously reported.^26^

Combination vaccines for diarrhoea are attractive^27^ because each pathogen accounts for only a modest proportion of the total burden of diarrhoea (eg, 10%),^28^ such that a strategy to implement multiple individual vaccines is unlikely to be desirable. Adding multiple immunisations, possibly with their own unique administration schedules, in an already crowded Expanded Programme on Immunization schedule is probably infeasible. However, few combination vaccines, except *Shigella*–ETEC vaccines,^29^ are currently in development. This analysis provides further evidence to support the development of combination vaccines and their value proposition.

Furthermore, these results suggest that other interventions to prevent diarrhoea, including water, sanitation, and hygiene interventions, could also be important to reduce the impact of diarrhoea-related antibiotic use and associated antimicrobial resistance, because WASH interventions could prevent diarrhoea due to multiple pathogens, and we note prevention of multiple pathogens simultanously is important for vaccine impact (ie, combination vaccines that prevent multiple pathogens are needed to make substantial impact. A recent modelling study found that water, sanitation, and hygiene interventions would be expected to have slightly larger impact on antimicrobial resistance related mortality in LMICs than universal access to paediatric vaccines.^30^

A *Campylobacter* vaccine was predicted to have minimal impact on antibiotic use, since few diarrhoea episodes were attributed to *Campylobacter*.^5^ In addition, the burden of *Campylobacter* is high in the first 6 months of life, such that a vaccine given at age 6 months and 9 months would miss a substantial proportion of *Campylobacter* episodes. However, these results were sensitive to the methods for attributing diarrhoea causation. Because *Campylobacter* is commonly detected at low quantities during diarrhoea, a *Campylobacter* vaccine was expected to be similarly effective for preventing antibiotic use as a rotavirus vaccine when diarrhoea cause was defined by detection at any quantity. The true burden of antibiotic use preventable by a *Campylobacter* vaccine might fall between these two extremes. Other vaccine impacts were less sensitive to the methods for defining diarrhoea causation.

This study was limited by the assumptions made about dosing schedules and vaccine efficacies for each of the vaccines, which were made based on distribution of disease, probable combination vaccines, and WHO preferred product characteristics.^18^, ^19^ Furthermore, the results might not be globally generalisable as there was considerable heterogeneity in impact across sites, and the primary results were based on diarrhoea incidence and causation distributions observed in the MAL-ED study, which concluded 10 years ago. Although newer multisite community-based studies of diarrhoea causation have not been conducted since MAL-ED, data from the Global Pediatric Diarrhoea Surveillance System suggests the distributions of causes of hospitalisations due to diarrhoea did not change between 2017 and 2021, despite the COVID-19 pandemic (excluding reductions in rotavirus due to introduction of a vaccine).^31^ When we incorporated estimated reductions in diarrhoea incidence between 2013 and 2021^25^ in a sensitivity analysis, the projected vaccine effects in 2021 were substantially lower than those during the MAL-ED study period. The impact of vaccines for diarrhoea on antibiotic use are expected to continue to become more limited if current declining trends in incidence continue, and as other interventions to prevent diarrhoeal diseases are implemented.

The primary results should be interpreted as an upper bound on vaccine impact since we assumed 100% vaccination coverage in all scenarios. The sensitivity analysis showed that the decrement in vaccine effect under a more realistic vaccination coverage scenario was proportional to the assumed coverage, suggesting coverage is a major determinant of vaccine impact. Furthermore, we were unable to confirm that diarrhoea was the indication for antibiotic treatment beyond the overlap with diarrhoea symptoms, which might have resulted in an overestimation of the antibiotic courses that could be prevented by diarrhoea targeted interventions. Alternatively, greater reductions could be realised if there are appreciable indirect effects of the vaccines, which were not considered here. Finally, it is unknown what absolute or relative magnitude of reduction in antibiotic use is necessary to translate to reductions in observed antimicrobial resistance.

In summary, an important additional component of the value proposition for vaccines for diarrhoea is the expected impact on reductions in antibiotic use, including inappropriate use for viral diarrhoea episodes. Although the absolute number of prevented antibiotic uses is expected to be considerable if enteric vaccines achieve 100% coverage, even realistic combination vaccines would have modest impact on total antibiotic use given the frequency of illness and treatment in children younger than 5 years in low-resource settings.

## Contributors

## Data sharing

De-identified participant data from the MAL-ED study is publicly available at ClinEpiDB.org after approval of a proposal by the study principal investigators. https://github.com/sb2yb/wellcome-vaccineproject.

## Declaration of interests

We declare no competing interests.
