# Supplementary material for: The impact of vaccines for diarrhoea on antibiotic use among children in five low-resource settings: a comparative simulation study
Source: Lancet Glob Health. 2024 Nov 20;12(12):e1954–61. doi: 10.1016/S2214-109X(24)00378-4 (PMC11584313; doi:10.1016/S2214-109X(24)00378-4)

THE LANCET
Global Health

Supplementary appendix

This appendix formed part of the original submission and has been peer reviewed. We post it as supplied by the authors.

Supplement to: Rogawski McQuade ET, Brennhofer SA, Elwood SE, et al. The impact of vaccines for diarrhoea on antibiotic use among children in five low-resource settings: a comparative simulation study. *Lancet Glob Health* 2024; **12:** e1954–61.

Supplementary appendix

The impact of vaccines for diarrhea on antibiotic use among children in low-resource settings: a comparative simulation study

Elizabeth T Rogawski McQuade; Stephanie A Brennhofer; Sarah E. Elwood; Joseph A Lewnard; Jie Liu; Eric R Houpt; James A Platts-Mills

Correspondence to: Elizabeth T Rogawski McQuade

Supplementary Materials

Table S1. Enteric vaccination scenarios simulated in the MAL-ED dataset, including dosing schedules and efficacies. 4

Supplementary Methods 5

Figure S1. Country-level incidence of all-cause diarrhea per 100,000 children under 2 years of age from 2009 to 2021 as estimated by the Global Burden of Disease Study from the Institute for Health Metrics and Evaluation. Selected countries match the five included sites of the MAL-ED birth cohort study. 6

Table S2. Ratio of all-cause diarrhea incidence in 2021 compared to that in 2013 as estimated by the Global Burden of Disease Study from the Institute for Health Metrics and Evaluation. Selected countries match the five included sites of the MAL-ED birth cohort study. 7

Table S3. Absolute (incidence rate differences) and relative (incidence rate ratios) differences and percent reductions in antibiotic use outcomes (any drug class) for nine enteric pathogen vaccine scenarios compared to the no vaccine scenario with 60% full vaccine efficacies against severe etiology-specific diarrhea at five sites in the MAL-ED birth cohort study. 8

Table S4. Absolute (incidence rate differences) and relative (incidence rate ratios) differences and percent reductions in fluroquinolone and macrolide (F/M) use outcomes for nine enteric pathogen vaccine scenarios compared to the no vaccine scenario with 60% full vaccine efficacies against severe etiology-specific diarrhea at five sites in the MAL-ED birth cohort study. 10

Figure S2. Absolute differences (incidence rate differences) in fluroquinolone and macrolide (F/M) use outcomes for nine enteric pathogen vaccine scenarios compared to the no vaccine scenario with 60% full vaccine efficacies against severe etiology-specific diarrhea at five sites in the MAL-ED birth cohort study. 12

Figure S3. Percent reductions in fluoroquinolone and macrolide (F/M) use outcome for nine enteric pathogen vaccine scenarios compared to the no vaccine scenario with 60% full vaccine efficacies against severe etiology-specific diarrhea at five sites in the MAL-ED birth cohort study. 13

Table S5. Absolute (incidence rate differences) and relative (incidence rate ratios) differences and percent reductions in antibiotic courses for diarrhea episodes of any etiology (any drug class) for nine enteric pathogen vaccine scenarios compared to the no vaccine scenario with 60% full vaccine efficacies against severe etiology-specific diarrhea at five sites in the MAL-ED birth cohort study. 14

Figures S4. Absolute differences (incidence rate differences) in antibiotic courses for diarrhea episodes of any etiology (any drug class) for nine enteric pathogen vaccine scenarios compared to the no vaccine scenario with 60% full vaccine efficacies against severe etiology-specific diarrhea at five sites in the MAL-ED birth cohort study. 16

Table S6. Sensitivity Analysis: Assuming country-specific diarrhea incidence rates for 2021 based on the Global Burden of Disease Study, absolute (incidence rate differences) and relative (incidence rate ratios) differences and percent reductions in antibiotic courses for diarrhea episodes of any etiology (any drug class) for nine enteric pathogen vaccine scenarios compared to the no vaccine scenario with 60% full vaccine efficacies against severe etiology-specific diarrhea at five sites in the MAL-ED birth cohort study. 17

Figures S5. Sensitivity Analysis: Assuming country-specific diarrhea incidence rates for 2021 based on the Global Burden of Disease Study, absolute (incidence rate differences) in antibiotic courses for diarrhea episodes of any etiology (any drug class) for nine enteric pathogen vaccine scenarios compared

to the no vaccine scenario with 60% full vaccine efficacies against severe etiology-specific diarrhea at five sites in the MAL-ED birth cohort study. 19

Table S7. Incidence rates of etiologic specific diarrhea (N=7458 stools) based on AFe>0.5 and Ct<35 at five sites in the MAL-ED birth cohort study. 20

Table S8. Sensitivity Analysis: Absolute (incidence rate differences) and relative (incidence rate ratios) differences and percent reductions in antibiotic use outcomes (any drug class) for nine enteric pathogen vaccine scenarios compared to the no vaccine scenario with 60% full vaccine efficacies at five sites in the MAL-ED birth cohort study when diarrhea etiology was defined by the detection of a pathogen by qPCR at any quantity (quantification cycle threshold < 35). 21

Table S9. Sensitivity Analysis: Absolute (incidence rate differences) and relative (incidence rate ratios) differences and percent reductions in antibiotic use outcomes (any drug class) for nine enteric pathogen vaccine scenarios compared to the no vaccine scenario with 60% full vaccine efficacies against severe etiology-specific diarrhea at five sites in the MAL-ED birth cohort study with 60% vaccine coverage.... 23

Figure S6. Sensitivity Analysis: Absolute (incidence rate) differences in antibiotic use outcomes for any drug class for nine enteric pathogen vaccine scenarios compared to the no vaccine scenario with 60% full vaccine efficacies against severe etiology-specific diarrhoea at five sites in the MAL-ED birth cohort study with 60% vaccine coverage. 25

Figure S7. Sensitivity Analysis: Percent reductions in antibiotic use of any drug class for nine enteric pathogen vaccine scenarios compared to the no vaccine scenario with 60% full vaccine efficacies against severe etiology-specific diarrhoea at five sites in the MAL-ED birth cohort study with 60% vaccine coverage. 26

Table S1. Enteric vaccination scenarios simulated in the MAL-ED dataset, including dosing schedules and efficacies.

Dosing schedule	Diarrhea severity	Efficacy 14 days after 1st dose	Efficacy 14 days after 2nd dose
1st: 2 months	Severe	30%	60%
2nd: 4 months	Non-severe	20%	40%
1st: 6 months	Severe	30%	60%
2nd: 9 months	Non-severe	20%	40%

Viral vaccines (dosing schedule: Months 2 & 4): Rotavirus, Norovirus GII, Adenovirus 40/41, Norovirus + Rotavirus, Adenovirus 40/41 + Norovirus GII + Rotavirus
Bacterial vaccines (dosing schedule: Months 6 & 9): *Shigella*, Enterotoxigenic *Escherichia coli* (ETEC), *Campylobacter jejuni/coli*, *Shigella* + ETEC

Supplementary Methods

Projecting vaccine effects on antibiotic use outcomes in 2021

To account for changes in diarrhoea incidence since the MAL-ED study, we used data from the Global Burden of Disease Study (GBD) from the Institute for Health Metrics and Evaluation. Specifically, we obtained estimates for the incidence of all-cause diarrhea among children aged <1 year and 12-23 months from the 5 countries with participating MAL-ED sites (Bangladesh, India, Nepal, Pakistan, and Tanzania) for all years between 2009 and 2021 (https://vizhub.healthdata.org/gbd-results/; Figure S1). Based on the confidence intervals provided for each estimate, we calculated expected standard errors and sampled 1000 draws for each incidence point estimate assuming a Poisson distribution. Incidence for the combined age group of children under 2 years was estimated by pairing and averaging draws from the <1 year and 12-23 month age groups. For each draw, we then fit a linear regression model for country-level incidence by calendar year to estimate the trend in incidence from 2009-2021. For all sites except Tanzania, a linear trend had the best fit; for Tanzania, year was modeled as a cubic term to capture non-linearities (i.e., two inflection points) in the trend. The ratio of incidence in 2021 (most recent estimates from GBD) relative to 2013 (mid-point of MAL-ED follow-up) was estimated by taking the ratio of the predicted incidences for those years from the regression model. The median, 2.5^{th}, and 97.5^{th} percentiles from the 1000 draws were used to construct point estimates and 95% confidence intervals (Table S2). To estimate projected vaccine effects on antibiotic use outcomes in 2021, we reduced the observed incidence of antibiotic treated diarrhoea at each site in proportion to the ratio of all-cause diarrhoea incidence from 2021 to 2013 for the corresponding country. All other methods for estimating vaccine effects were the same as primary analyses.

Figure S1. Country-level incidence of all-cause diarrhea per 100,000 children under 2 years of age from 2009 to 2021 as estimated by the Global Burden of Disease Study from the Institute for Health Metrics and Evaluation. Selected countries match the five included sites of the MAL-ED birth cohort study.

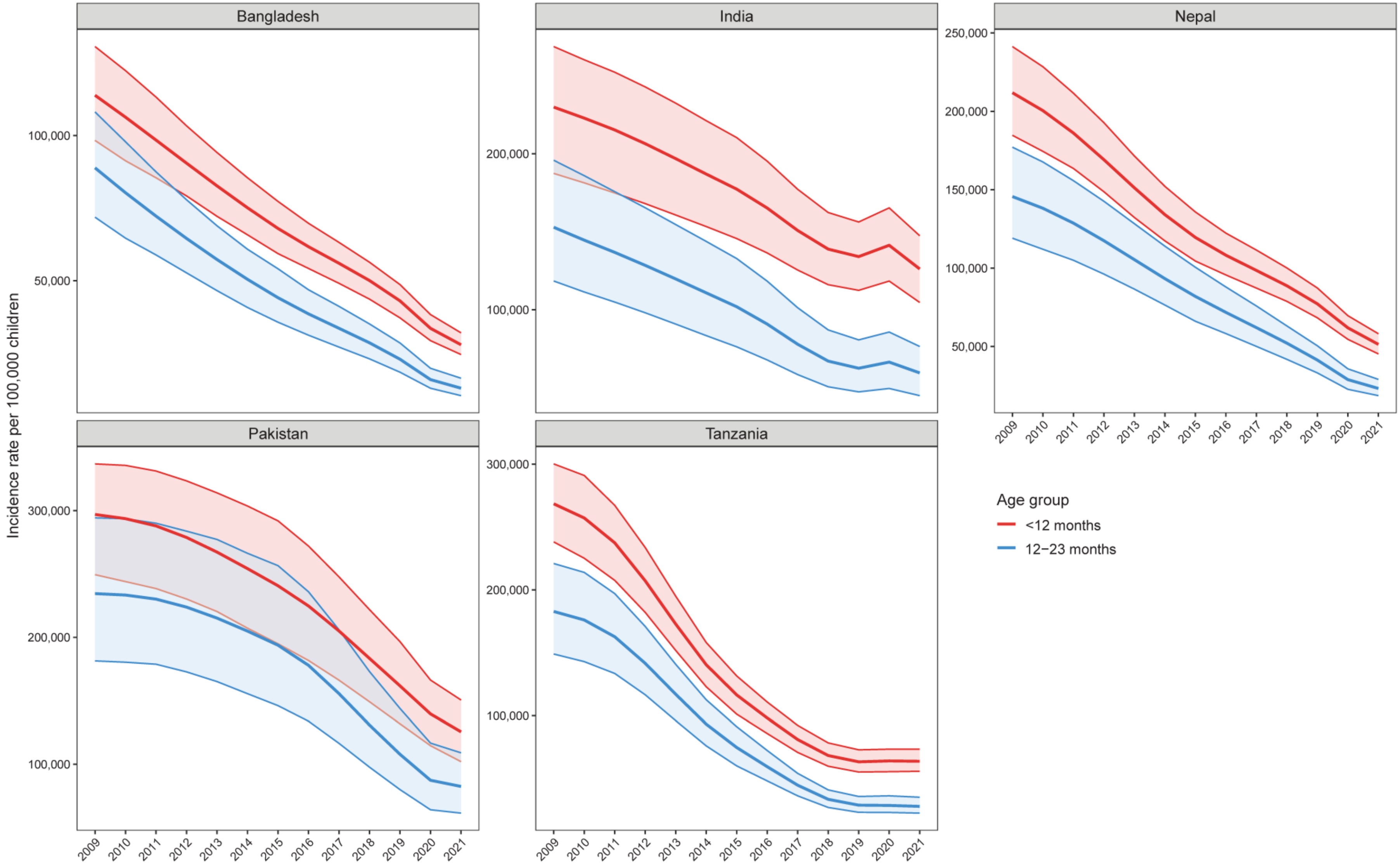

Source: https://vizhub.healthdata.org/gbd-results/

Table S2. Ratio of all-cause diarrhea incidence in 2021 compared to that in 2013 as estimated by the Global Burden of Disease Study from the Institute for Health Metrics and Evaluation. Selected countries match the five included sites of the MAL-ED birth cohort study.

**Country**	**Ratio (95% CI)**
Bangladesh	0.26 (0.21, 0.30)
India	0.55 (0.48, 0.62)
Nepal	0.26 (0.22, 0.30)
Pakistan	0.49 (0.44, 0.56)
Tanzania	0.34 (0.31, 0.38)

Source: https://vizhub.healthdata.org/gbd-results/

Table S3. Absolute (incidence rate differences) and relative (incidence rate ratios) differences and percent reductions in antibiotic use outcomes (any drug class) for nine enteric pathogen vaccine scenarios compared to the no vaccine scenario with 60% full vaccine efficacies against severe etiology-specific diarrhea at five sites in the MAL-ED birth cohort study.

Vaccine scenario and efficacy outcome	Incidence rate difference (cases per 100 child years)	Incidence rate ratio	Percent reduction
Rotavirus			
Antibiotic courses for bacterial diarrhea episodes	-1·0 (-1·3, -0·6)	0·97 (0·96, 0·98)	-2·8 (-3·8, -2·0)
Antibiotic courses for viral/parasitic diarrhea episodes	-6·6 (-7·5, -5·7)	0·86 (0·84, 0·88)	-14·1 (-15·7, -12·4)
Antibiotic courses for diarrhea episodes of any etiology	-6·6 (-7·5, -5·7)	0·95 (0·94, 0·96)	-4·9 (-5·6, -4·2)
Antibiotic courses overall	-6·6 (-7·5, -5·7)	0·99 (0·99, 0·99)	-0·8 (-1·0, -0·7)
Antibiotic exposures to bystander pathogens overall	-12·0 (-14·2, -9·9)	0·99 (0·99, 0·99)	-0·9 (-1·1, -0·8)
Norovirus GII			
Antibiotic courses for bacterial diarrhea episodes	-0·5 (-0·8, -0·3)	0·98 (0·98, 0·99)	-1·5 (-2·3, -0·9)
Antibiotic courses for viral/parasitic diarrhea episodes	-1·7 (-2·2, -1·3)	0·96 (0·95, 0·97)	-3·6 (-4·6, -2·8)
Antibiotic courses for diarrhea episodes of any etiology	-1·7 (-2·2, -1·3)	0·99 (0·98, 0·99)	-1·3 (-1·6, -1·0)
Antibiotic courses overall	-1·7 (-2·2, -1·3)	--	-0·2 (-0·3, -0·2)
Antibiotic exposures to bystander pathogens overall	-3·1 (-4·2, -2·2)	--	-0·2 (-0·3, -0·2)
Adenovirus 40/41			
Antibiotic courses for bacterial diarrhea episodes	-2·1 (-2·7, -1·6)	0·94 (0·92, 0·95)	-6·3 (-7·8, -5·0)
Antibiotic courses for viral/parasitic diarrhea episodes	-4·8 (-5·8, -3·9)	0·90 (0·88, 0·91)	-10·3 (-11·9, -8·8)
Antibiotic courses for diarrhea episodes of any etiology	-4·8 (-5·8, -3·9)	0·96 (0·96, 0·97)	-3·6 (-4·3, -3·0)
Antibiotic courses overall	-4·8 (-5·8, -3·9)	0·99 (0·99, 1·00)	-0·6 (-0·7, -0·5)
Antibiotic exposures to bystander pathogens overall	-10·1 (-12·3, -8·2)	0·99 (0·99, 0·99)	-0·8 (-0·9, -0·6)
Norovirus GII + Rotavirus			
Antibiotic courses for bacterial diarrhea episodes	-1·4 (-1·9, -1·1)	0·96 (0·95, 0·97)	-4·3 (-5·4, -3·2)
Antibiotic courses for viral/parasitic diarrhea episodes	-8·1 (-9·2, -7·1)	0·83 (0·81, 0·85)	-17·3 (-19·1, -15·5)
Antibiotic courses for diarrhea episodes of any etiology	-8·1 (-9·2, -7·1)	0·94 (0·93, 0·95)	-6·0 (-6·8, -5·3)
Antibiotic courses overall	-8·1 (-9·2, -7·1)	0·99 (0·99, 0·99)	-1·0 (-1·2, -0·9)
Antibiotic exposures to bystander pathogens overall	-14·7 (-17·1, -12·2)	0·99 (0·99, 0·99)	-1·1 (-1·3, -0·9)
Adenovirus 40/41 + Norovirus GII + Rotavirus			
Antibiotic courses for bacterial diarrhea episodes	-3·4 (-4·0, -2·7)	0·90 (0·88, 0·92)	-10·0 (-11·6, -8·3)
Antibiotic courses for viral/parasitic diarrhea episodes	-12·2 (-13·7, -11·0)	0·74 (0·72, 0·76)	-26·1 (-27·9, -24·1)
Antibiotic courses for diarrhea episodes of any etiology	-12·2 (-13·7, -11·0)	0·91 (0·90, 0·92)	-9·1 (-10·1, -8·1)
Antibiotic courses overall	-12·2 (-13·7, -11·0)	0·98 (0·98, 0·99)	-1·5 (-1·7, -1·4)
Antibiotic exposures to bystander pathogens overall	-23·1 (-26·5, -20·2)	0·98 (0·98, 0·98)	-1·8 (-2·0, -1·6)
Shigella			
Antibiotic courses for bacterial diarrhea episodes	-8·2 (-9·4, -7·2)	0·75 (0·73, 0·77)	-24·5 (-26·6, -22·6)

Antibiotic courses for viral/parasitic diarrhea episodes	-3·1 (-3·7, -2·4)	0·93 (0·92, 0·95)	-6·6 (-7·8, -5·3)
Antibiotic courses for diarrhea episodes of any etiology	-8·2 (-9·4, -7·2)	0·94 (0·93, 0·95)	-6·1 (-6·9, -5·3)
Antibiotic courses overall	-8·2 (-9·4, -7·2)	0·99 (0·99, 0·99)	-1·0 (-1·2, -0·9)
Antibiotic exposures to bystander pathogens overall	-15·9 (-18·5, -13·8)	0·99 (0·99, 0·99)	-1·2 (-1·4, -1·1)
Enterotoxigenic *Escherichia coli* (ETEC)			
Antibiotic courses for bacterial diarrhea episodes	-4·4 (-5·2, -3·7)	0·87 (0·85, 0·89)	-13·2 (-15·0, -11·5)
Antibiotic courses for viral/parasitic diarrhea episodes	-1·8 (-2·2, -1·4)	0·96 (0·95, 0·97)	-3·8 (-4·8, -3·0)
Antibiotic courses for diarrhea episodes of any etiology	-4·4 (-5·2, -3·7)	0·97 (0·96, 0·97)	-3·3 (-3·8, -2·8)
Antibiotic courses overall	-4·4 (-5·2, -3·7)	0·99 (0·99, 1·00)	-0·6 (-0·7, -0·5)
Antibiotic exposures to bystander pathogens overall	-7·8 (-9·4, -6·2)	0·99 (0·99, 1·00)	-0·6 (-0·7, -0·5)
Campylobacter jejuni/coli			
Antibiotic courses for bacterial diarrhea episodes	-0·2 (-0·3, 0·0)	1·00 (0·99, 1·00)	-0·5 (-0·9, -0·1)
Antibiotic courses for viral/parasitic diarrhea episodes	0·0 (-0·1, 0·0)	--	0·0 (-0·2, 0·0)
Antibiotic courses for diarrhea episodes of any etiology	-0·2 (-0·3, 0·0)	--	-0·1 (-0·2, 0·0)
Antibiotic courses overall	-0·2 (-0·3, 0·0)	--	0·0 (0·0, 0·0)
Antibiotic exposures to bystander pathogens overall	-0·3 (-0·6, -0·1)	--	0·0 (0·0, 0·0)
Shigella + ETEC			
Antibiotic courses for bacterial diarrhea episodes	-11·3 (-12·7, -10·2)	0·66 (0·64, 0·68)	-33·8 (-35·7, -32·1)
Antibiotic courses for viral/parasitic diarrhea episodes	-4·4 (-5·2, -3·7)	0·91 (0·89, 0·92)	-9·5 (-10·8, -8·2)
Antibiotic courses for diarrhea episodes of any etiology	-11·3 (-12·7, -10·2)	0·92 (0·91, 0·92)	-8·4 (-9·3, -7·6)
Antibiotic courses overall	-11·3 (-12·7, -10·2)	0·99 (0·98, 0·99)	-1·4 (-1·6, -1·3)
Antibiotic exposures to bystander pathogens overall	-21·3 (-24·5, -18·7)	0·98 (0·98, 0·99)	-1·7 (-1·9, -1·5)

Viral vaccines administered at 2 and 4 months. Bacterial vaccines administered at 6 and 9 months.
Data also reported in Figures 1 and 2.

Table S4. Absolute (incidence rate differences) and relative (incidence rate ratios) differences and percent reductions in fluroquinolone and macrolide (F/M) use outcomes for nine enteric pathogen vaccine scenarios compared to the no vaccine scenario with 60% full vaccine efficacies against severe etiology-specific diarrhea at five sites in the MAL-ED birth cohort study.

Vaccine scenario and efficacy outcome	Incidence rate difference (cases per 100 child years)	Incidence rate ratio	Percent reduction
Rotavirus			
F/M courses for bacterial diarrhea episodes	-0·6 (-0·9, -0·4)	0·96 (0·94, 0·97)	-4·1 (-6·0, -2·6)
F/M courses for viral/parasitic diarrhea episodes	-3·1 (-3·8, -2·5)	0·85 (0·82, 0·87)	-15·4 (-17·7, -13·3)
F/M courses for diarrhea episodes of any etiology	-3·1 (-3·8, -2·5)	0·93 (0·91, 0·94)	-7·5 (-8·7, -6·3)
F/M courses overall	-3·1 (-3·8, -2·5)	0·98 (0·97, 0·98)	-2·3 (-2·8, -2·0)
F/M exposures to bystander pathogens overall	-6·4 (-8·1, -5·1)	0·97 (0·97, 0·98)	-2·5 (-3·1, -2·1)
Norovirus GII			
F/M courses for bacterial diarrhea episodes	-0·1 (-0·3, 0·0)	0·99 (0·98, 1·00)	-0·9 (-1·9, -0·3)
F/M courses for viral/parasitic diarrhea episodes	-0·5 (-0·7, -0·3)	0·98 (0·97, 0·98)	-2·4 (-3·4, -1·5)
F/M courses for diarrhea episodes of any etiology	-0·5 (-0·7, -0·3)	0·99 (0·98, 0·99)	-1·2 (-1·7, -0·7)
F/M courses overall	-0·5 (-0·7, -0·3)	1·00 (0·99, 1·00)	-0·4 (-0·5, -0·2)
F/M exposures to bystander pathogens overall	-1·0 (-1·5, -0·6)	1·00 (0·99, 1·00)	-0·4 (-0·6, -0·2)
Adenovirus 40/41			
F/M courses for bacterial diarrhea episodes	-1·5 (-2·0, -1·1)	0·90 (0·88, 0·92)	-9·9 (-12·3, -7·7)
F/M courses for viral/parasitic diarrhea episodes	-3·1 (-3·8, -2·5)	0·85 (0·82, 0·87)	-15·5 (-17·8, -13·4)
F/M courses for diarrhea episodes of any etiology	-3·1 (-3·8, -2·5)	0·92 (0·91, 0·94)	-7·5 (-8·7, -6·3)
F/M courses overall	-3·1 (-3·8, -2·5)	0·98 (0·97, 0·98)	-2·4 (-2·8, -1·9)
F/M exposures to bystander pathogens overall	-6·7 (-8·3, -5·3)	0·97 (0·97, 0·98)	-2·7 (-3·2, -2·2)
Norovirus GII + Rotavirus			
F/M courses for bacterial diarrhea episodes	-0·7 (-1·1, -0·5)	0·95 (0·93, 0·97)	-4·9 (-7·0, -3·2)
F/M courses for viral/parasitic diarrhea episodes	-3·6 (-4·3, -3·0)	0·82 (0·80, 0·85)	-17·6 (-20·1, -15·3)
F/M courses for diarrhea episodes of any etiology	-3·6 (-4·3, -3·0)	0·91 (0·90, 0·93)	-8·5 (-10·0, -7·2)
F/M courses overall	-3·6 (-4·3, -3·0)	0·97 (0·97, 0·98)	-2·7 (-3·2, -2·2)
F/M exposures to bystander pathogens overall	-7·3 (-9·1, -5·8)	0·97 (0·96, 0·98)	-2·9 (-3·5, -2·4)
Adenovirus 40/41 + Norovirus GII + Rotavirus			
F/M courses for bacterial diarrhea episodes	-2·0 (-2·6, -1·6)	0·86 (0·83, 0·89)	-13·7 (-16·5, -11·1)
F/M courses for viral/parasitic diarrhea episodes	-6·2 (-7·3, -5·3)	0·7 (0·67, 0·72)	-30·4 (-32·8, -28·0)
F/M courses for diarrhea episodes of any etiology	-6·2 (-7·3, -5·3)	0·85 (0·84, 0·87)	-14·7 (-16·5, -13·1)
F/M courses overall	-6·2 (-7·3, -5·3)	0·95 (0·95, 0·96)	-4·6 (-5·3, -4·1)
F/M exposures to bystander pathogens overall	-12·8 (-15·4, -10·6)	0·95 (0·94, 0·96)	-5·1 (-5·8, -4·4)
Shigella			
F/M courses for bacterial diarrhea episodes	-4·0 (-4·7, -3·2)	0·73 (0·71, 0·76)	-26·6 (-29·1, -24·1)

F/M courses for viral/parasitic diarrhea episodes	-1·9 (-2·4, -1·5)	0·91 (0·88, 0·93)	-9·3 (-11·5, -7·4)
F/M courses for diarrhea episodes of any etiology	-4·0 (-4·7, -3·2)	0·91 (0·89, 0·92)	-9·5 (-10·8, -8·1)
F/M courses overall	-4·0 (-4·7, -3·2)	0·97 (0·97, 0·97)	-3·0 (-3·4, -2·5)
F/M exposures to bystander pathogens overall	-8·5 (-10·2, -6·9)	0·97 (0·96, 0·97)	-3·4 (-3·9, -2·8)
Enterotoxigenic *Escherichia coli* (ETEC)			
F/M courses for bacterial diarrhea episodes	-2·2 (-2·7, -1·7)	0·85 (0·83, 0·88)	-14·7 (-17·0, -12·4)
F/M courses for viral/parasitic diarrhea episodes	-0·9 (-1·2, -0·6)	0·96 (0·94, 0·97)	-4·3 (-5·6, -3·1)
F/M courses for diarrhea episodes of any etiology	-2·2 (-2·7, -1·7)	0·95 (0·94, 0·96)	-5·2 (-6·1, -4·3)
F/M courses overall	-2·2 (-2·7, -1·7)	0·98 (0·98, 0·99)	-1·6 (-2, -1·3)
F/M exposures to bystander pathogens overall	-3·8 (-4·9, -2·8)	0·99 (0·98, 0·99)	-1·5 (-1·9, -1·1)
Campylobacter jejuni/coli			
F/M courses for bacterial diarrhea episodes	0·0 (-0·1, 0·0)	--	-0·1 (-0·5, 0·0)
F/M courses for viral/parasitic diarrhea episodes	0·0 (-0·1, 0·0)	--	-0·1 (-0·3, 0·0)
F/M courses for diarrhea episodes of any etiology	0·0 (-0·1, 0·0)	--	0·0 (-0·2, 0·0)
F/M courses overall	0·0 (-0·1, 0·0)	--	0·0 (-0·1, 0·0)
F/M exposures to bystander pathogens overall	0·0 (-0·2, 0·0)	--	0·0 (-0·1, 0·0)
Shigella + ETEC			
F/M courses for bacterial diarrhea episodes	-5·4 (-6·3, -4·5)	0·64 (0·62, 0·66)	-36·2 (-38·2, -34·2)
F/M courses for viral/parasitic diarrhea episodes	-2·5 (-3·1, -2·0)	0·88 (0·85, 0·89)	-12·4 (-14·5, -10·6)
F/M courses for diarrhea episodes of any etiology	-5·4 (-6·3, -4·5)	0·87 (0·86, 0·88)	-12·9 (-14·4, -11·5)
F/M courses overall	-5·4 (-6·3, -4·5)	0·96 (0·95, 0·96)	-4·1 (-4·6, -3·5)
F/M exposures to bystander pathogens overall	-11·1 (-13·3, -9·0)	0·96 (0·95, 0·96)	-4·4 (-5·0, -3·7)

Viral vaccines administered at 2 and 4 months. Bacterial vaccines administered at 6 and 9 months.
Data also reported in Figures S2 and S3.

Figure S2. Absolute differences (incidence rate differences) in fluroquinolone and macrolide (F/M) use outcomes for nine enteric pathogen vaccine scenarios compared to the no vaccine scenario with 60% full vaccine efficacies against severe etiology-specific diarrhea at five sites in the MAL-ED birth cohort study.

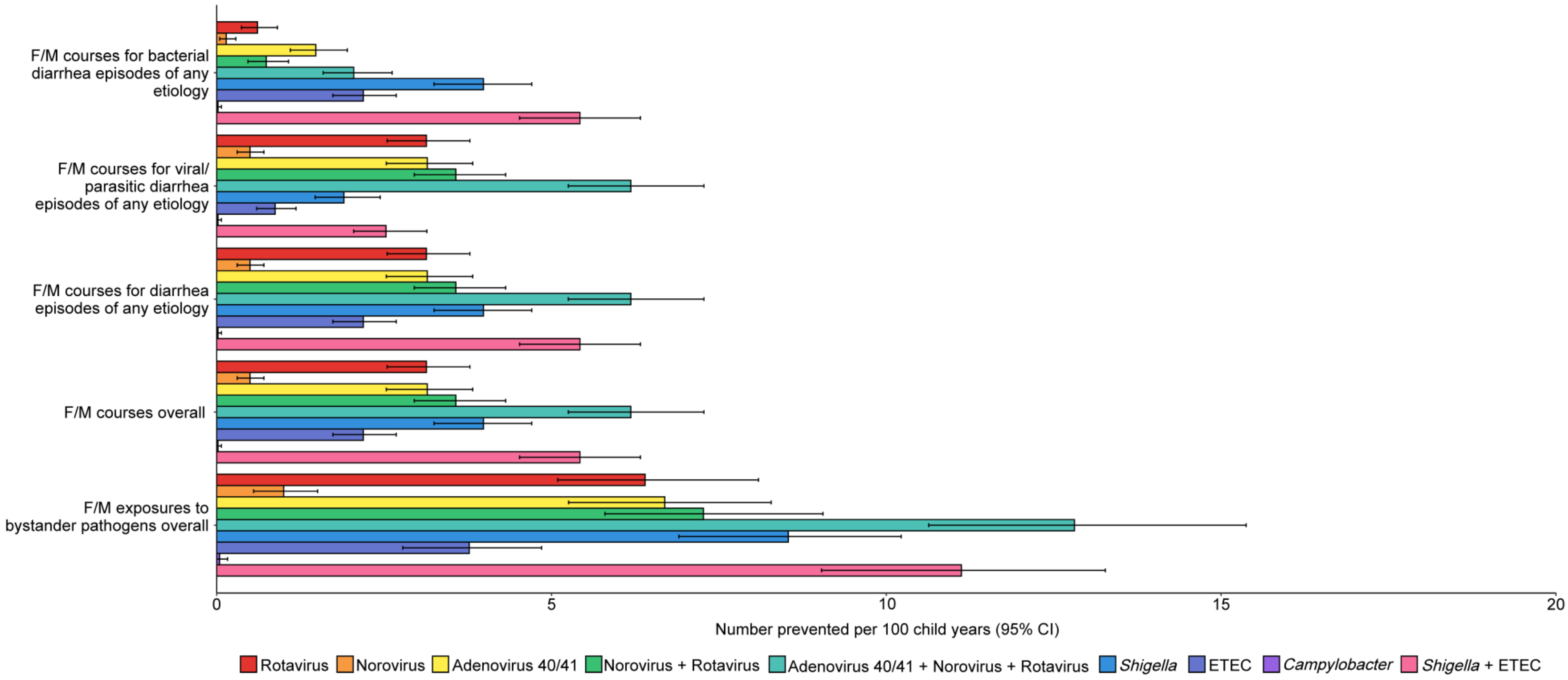

Figure S3. Percent reductions in fluoroquinolone and macrolide (F/M) use outcome for nine enteric pathogen vaccine scenarios compared to the no vaccine scenario with 60% full vaccine efficacies against severe etiology-specific diarrhea at five sites in the MAL-ED birth cohort study.

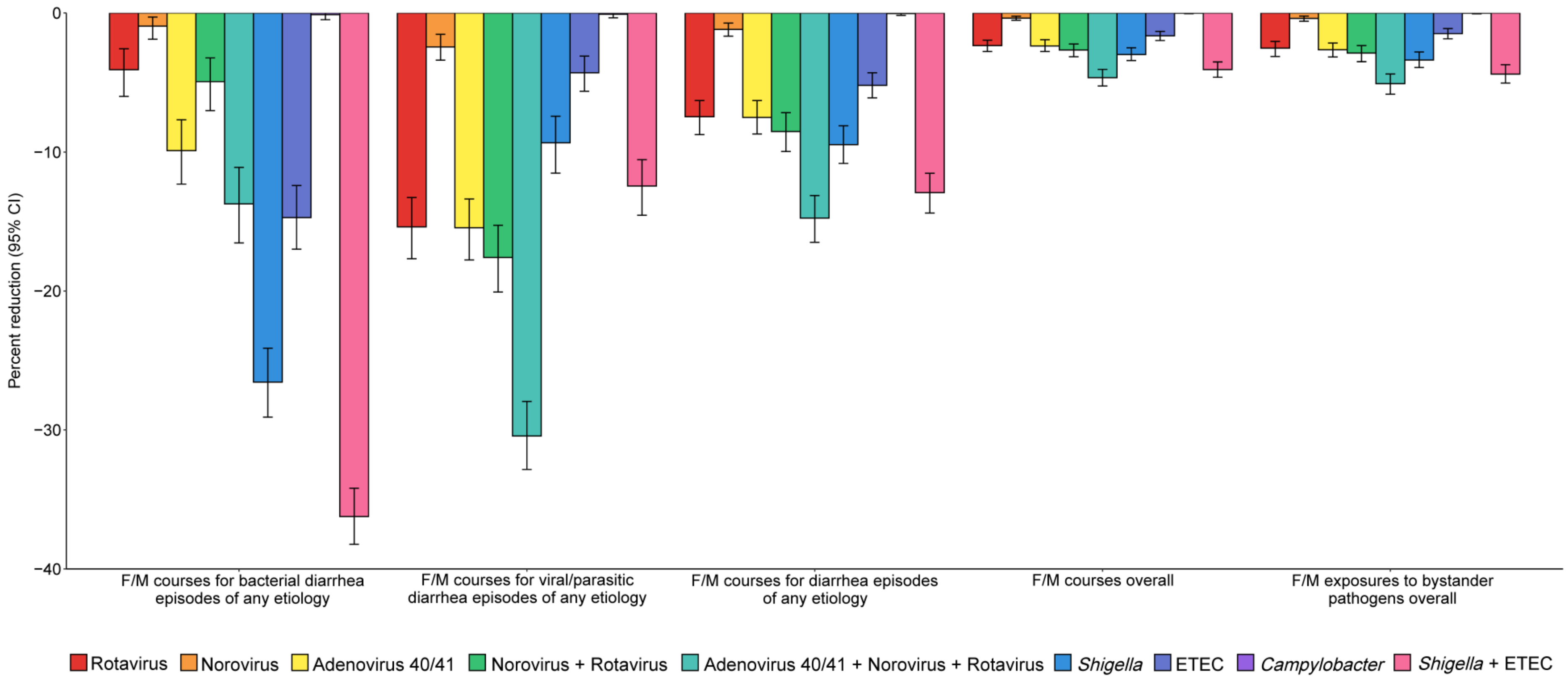

Table S5. Absolute (incidence rate differences) and relative (incidence rate ratios) differences and percent reductions in antibiotic courses for diarrhea episodes of any etiology (any drug class) for nine enteric pathogen vaccine scenarios compared to the no vaccine scenario with 60% full vaccine efficacies against severe etiology-specific diarrhea at five sites in the MAL-ED birth cohort study.

Vaccine scenario and efficacy outcome	Incidence rate difference (cases per 100 child years)	Incidence rate ratio	Percent reduction
Bangladesh			
Rotavirus	-2·8 (-3·5, -2·2)	0·93 (0·91, 0·94)	-7·3 (-8·6, -6·2)
Norovirus GII	-0·6 (-0·8, -0·4)	0·99 (0·98, 0·99)	-1·5 (-2·0, -1·0)
Adenovirus 40/41	-3·4 (-4·1, -2·7)	0·91 (0·90, 0·92)	-8·8 (-10·2, -7·5)
Norovirus GII + Rotavirus	-3·3 (-4·1, -2·6)	0·91 (0·90, 0·93)	-8·6 (-10·1, -7·2)
Adenovirus 40/41 + Norovirus GII + Rotavirus	-6·2 (-7·3, -5·1)	0·84 (0·82, 0·86)	-15·9 (-17·8, -14·3)
Shigella	-3·8 (-4·5, -3·0)	0·90 (0·89, 0·92)	-9·7 (-11·2, -8·4)
Enterotoxigenic *Escherichia coli* (ETEC)	-2·2 (-2·7, -1·7)	0·94 (0·93, 0·95)	-5·6 (-6·6, -4·7)
Campylobacter jejuni/coli	0·0 (-0·1, 0·0)	--	0·0 (-0·2, 0·0)
Shigella + ETEC	-5·3 (-6·3, -4·4)	0·86 (0·85, 0·88)	-13·5 (-15·2, -12·1)
India			
Rotavirus	-0·7 (-1·0, -0·4)	0·93 (0·90, 0·96)	-7·0 (-10·1, -4·3)
Norovirus GII	0·0 (-0·1, 0·0)	1·00 (0·99, 1·00)	-0·5 (-1·4, 0·0)
Adenovirus 40/41	-0·2 (-0·4, -0·1)	0·97 (0·96, 0·99)	-2·5 (-4·4, -1·0)
Norovirus GII + Rotavirus	-0·7 (-1·1, -0·4)	0·93 (0·89, 0·95)	-7·5 (-10·8, -4·7)
Adenovirus 40/41 + Norovirus GII + Rotavirus	-1·0 (-1·4, -0·6)	0·90 (0·86, 0·93)	-10·0 (-13·7, -6·9)
Shigella	-0·8 (-1·2, -0·5)	0·92 (0·88, 0·94)	-8·4 (-11·6, -5·6)
Enterotoxigenic *Escherichia coli* (ETEC)	-0·3 (-0·5, -0·1)	0·97 (0·95, 0·99)	-3·3 (-5·3, -1·5)
Campylobacter jejuni/coli	-0·1 (-0·2, 0·0)	0·99 (0·98, 1·00)	-0·7 (-1·6, 0·0)
Shigella + ETEC	-1·0 (-1·4, -0·7)	0·89 (0·86, 0·93)	-10·6 (-14·2, -7·4)
Nepal			
Rotavirus	-0·5 (-0·8, -0·3)	0·96 (0·94, 0·97)	-4·1 (-5·9, -2·6)
Norovirus GII	-0·4 (-0·6, -0·2)	0·97 (0·95, 0·98)	-3·1 (-4·6, -1·7)
Adenovirus 40/41	-0·1 (-0·2, 0·0)	0·99 (0·99, 1·00)	-0·5 (-1·4, 0·0)
Norovirus GII + Rotavirus	-0·8 (-1·2, -0·6)	0·93 (0·91, 0·95)	-6·7 (-8·7, -4·8)
Adenovirus 40/41 + Norovirus GII + Rotavirus	-0·9 (-1·2, -0·6)	0·93 (0·91, 0·95)	-7·3 (-9·4, -5·3)
Shigella	-1·0 (-1·3, -0·7)	0·92 (0·89, 0·95)	-7·8 (-10·5, -5·5)
Enterotoxigenic *Escherichia coli* (ETEC)	-0·5 (-0·7, -0·3)	0·96 (0·95, 0·98)	-3·7 (-5·3, -2·1)
Campylobacter jejuni/coli	0·0 (0·0, 0·0)	--	0·0 (0·0, 0·0)
Shigella + ETEC	-1·2 (-1·6, -0·8)	0·90 (0·88, 0·93)	-9·6 (-12·2, -7·3)
Pakistan			
Rotavirus	-1·6 (-2·1, -1·1)	0·97 (0·97, 0·98)	-2·5 (-3·2, -1·8)
Norovirus GII	-0·3 (-0·6, -0·1)	1·00 (0·99, 1·00)	-0·5 (-1·0, -0·1)

Adenovirus 40/41	0·0 (0·0, 0·0)	--	0·0 (0·0, 0·0)
Norovirus GII + Rotavirus	-1·9 (-2·5, -1·4)	0·97 (0·96, 0·98)	-3·0 (-3·9, -2·2)
Adenovirus 40/41 + Norovirus GII + Rotavirus	-1·9 (-2·5, -1·4)	0·97 (0·96, 0·98)	-3·0 (-3·9, -2·2)
Shigella	-1·5 (-2·1, -1·1)	0·98 (0·97, 0·98)	-2·4 (-3·2, -1·8)
Enterotoxigenic *Escherichia coli* (ETEC)	-0·6 (-1·0, -0·3)	0·99 (0·98, 0·99)	-1·0 (-1·5, -0·5)
Campylobacter jejuni/coli	-0·1 (-0·2, 0·0)	--	-0·1 (-0·3, 0·0)
Shigella + ETEC	-2·0 (-2·7, -1·5)	0·97 (0·96, 0·98)	-3·2 (-4·2, -2·5)
Tanzania			
Rotavirus	-0·5 (-1·0, -0·2)	0·95 (0·92, 0·98)	-4·7 (-8·1, -1·8)
Norovirus GII	-0·5 (-0·8, -0·2)	0·96 (0·93, 0·98)	-4·1 (-6·9, -1·8)
Adenovirus 40/41	-0·2 (-0·5, -0·1)	0·98 (0·96, 0·99)	-2·1 (-4·3, -0·5)
Norovirus GII + Rotavirus	-1·0 (-1·5, -0·6)	0·91 (0·88, 0·95)	-8·7 (-12·4, -5·3)
Adenovirus 40/41 + Norovirus GII + Rotavirus	-1·2 (-1·7, -0·7)	0·90 (0·86, 0·93)	-10·3 (-14·4, -6·6)
Shigella	-0·2 (-0·5, 0·0)	0·98 (0·96, 1·00)	-2·0 (-4·3, -0·4)
Enterotoxigenic *Escherichia coli* (ETEC)	-0·6 (-1·0, -0·3)	0·95 (0·92, 0·97)	-5·1 (-8·1, -2·6)
Campylobacter jejuni/coli	0·0 (0·0, 0·0)	--	0·0 (0·0, 0·0)
Shigella + ETEC	-0·7 (-1·1, -0·4)	0·94 (0·91, 0·97)	-6·1 (-9·2, -3·4)

Viral vaccines administered at 2 and 4 months. Bacterial vaccines administered at 6 and 9 months.
Data also reported in Figures 3 and S4.

Figures S4. Absolute differences (incidence rate differences) in antibiotic courses for diarrhea episodes of any etiology (any drug class) for nine enteric pathogen vaccine scenarios compared to the no vaccine scenario with 60% full vaccine efficacies against severe etiology-specific diarrhea at five sites in the MAL-ED birth cohort study.

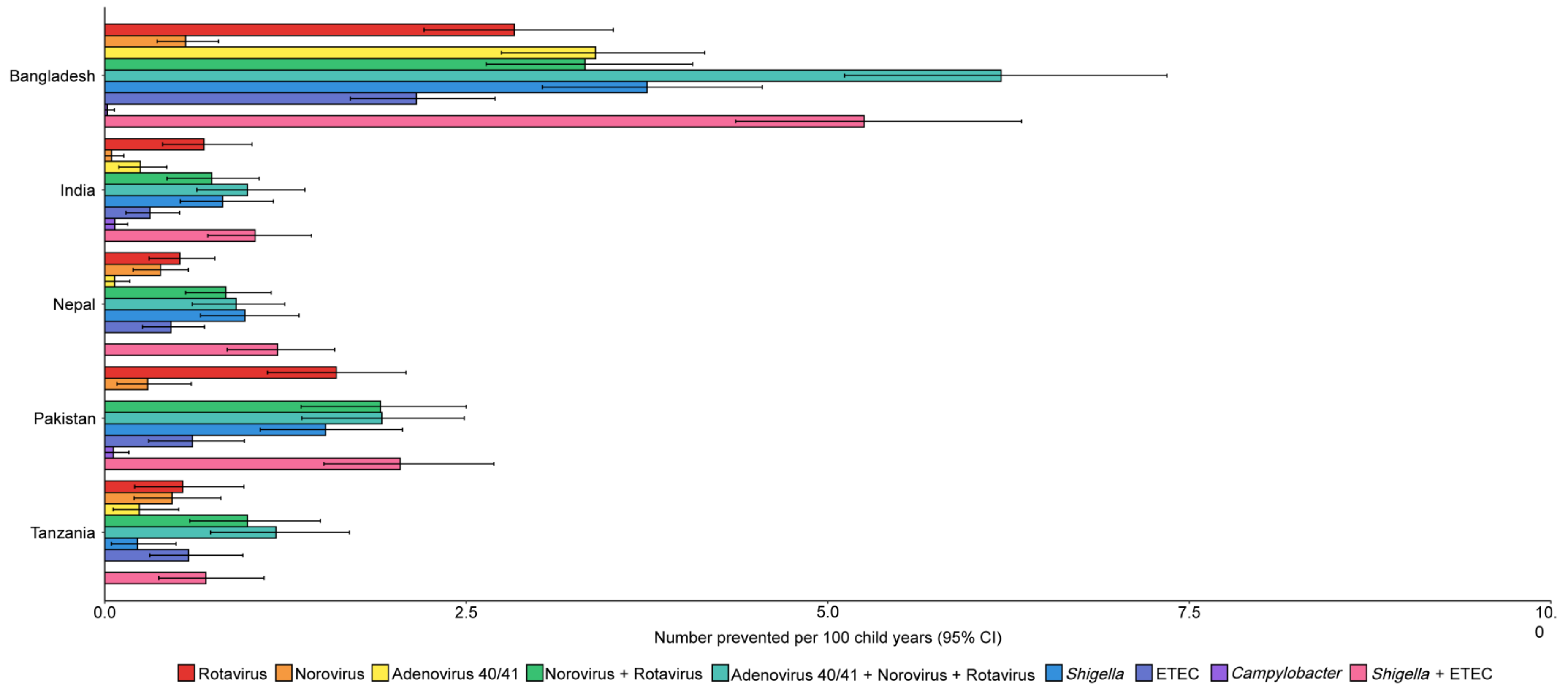

Table S6. Sensitivity Analysis: Assuming country-specific diarrhea incidence rates for 2021 based on the Global Burden of Disease Study, absolute (incidence rate differences) and relative (incidence rate ratios) differences and percent reductions in antibiotic courses for diarrhea episodes of any etiology (any drug class) for nine enteric pathogen vaccine scenarios compared to the no vaccine scenario with 60% full vaccine efficacies against severe etiology-specific diarrhea at five sites in the MAL-ED birth cohort study.

Vaccine scenario and efficacy outcome	Incidence rate difference (cases per 100 child years)	Incidence rate ratio	Percent reduction
Bangladesh			
Rotavirus	-0.7 (-0.9, -0.5)	0.93 (0.91, 0.94)	-7.3 (-8.6, -6.2)
Norovirus GII	-0.1 (-0.2, -0.1)	0.99 (0.98, 0.99)	-1.5 (-2.0, -1.0)
Adenovirus 40/41	-0.9 (-1.1, -0.6)	0.91 (0.90, 0.92)	-8.8 (-10.2, -7.5)
Norovirus GII + Rotavirus	-0.8 (-1.1, -0.6)	0.91 (0.90, 0.93)	-8.6 (-10.1, -7.2)
Adenovirus 40/41 + Norovirus GII + Rotavirus	-1.6 (-2.0, -1.2)	0.84 (0.82, 0.86)	-15.9 (-17.8, -14.3)
Shigella	-1.0 (-1.2, -0.7)	0.90 (0.89, 0.92)	-9.7 (-11.2, -8.4)
Enterotoxigenic *Escherichia coli* (ETEC)	-0.5 (-0.7, -0.4)	0.94 (0.93, 0.95)	-5.6 (-6.6, -4.7)
Campylobacter jejuni/coli	0.0 (0.0, 0.0)	--	0.0 (-0.2, 0.0)
Shigella + ETEC	-1.3 (-1.7, -1.0)	0.86 (0.85, 0.88)	-13.5 (-15.2, -12.1)
India			
Rotavirus	-0.4 (-0.6, -0.2)	0.93 (0.90, 0.96)	-7.0 (-10.1, -4.3)
Norovirus GII	0.0 (-0.1, 0.0)	1.00 (0.99, 1.00)	-0.5 (-1.4, 0.0)
Adenovirus 40/41	-0.1 (-0.2, -0.1)	0.97 (0.96, 0.99)	-2.5 (-4.4, -1.0)
Norovirus GII + Rotavirus	-0.4 (-0.6, -0.2)	0.93 (0.89, 0.95)	-7.5 (-10.8, -4.7)
Adenovirus 40/41 + Norovirus GII + Rotavirus	-0.5 (-0.8, -0.3)	0.90 (0.86, 0.93)	-10.0 (-13.7, -6.9)
Shigella	-0.4 (-0.6, -0.3)	0.92 (0.88, 0.94)	-8.4 (-11.6, -5.6)
Enterotoxigenic *Escherichia coli* (ETEC)	-0.2 (-0.3, -0.1)	0.97 (0.95, 0.99)	-3.3 (-5.3, -1.5)
Campylobacter jejuni/coli	0.0 (-0.1, 0.0)	0.99 (0.98, 1.00)	-0.7 (-1.6, 0.0)
Shigella + ETEC	-0.6 (-0.8, -0.4)	0.89 (0.86, 0.93)	-10.6 (-14.2, -7.4)
Nepal			
Rotavirus	-0.1 (-0.2, -0.1)	0.96 (0.94, 0.97)	-4.1 (-5.9, -2.6)
Norovirus GII	-0.1 (-0.2, 0.0)	0.97 (0.95, 0.98)	-3.1 (-4.6, -1.7)
Adenovirus 40/41	0.0 (0.0, 0.0)	0.99 (0.99, 1.00)	-0.5 (-1.4, 0.0)
Norovirus GII + Rotavirus	-0.2 (-0.3, -0.1)	0.93 (0.91, 0.95)	-6.7 (-8.7, -4.8)
Adenovirus 40/41 + Norovirus GII + Rotavirus	-0.2 (-0.3, -0.1)	0.93 (0.91, 0.95)	-7.3 (-9.4, -5.3)
Shigella	-0.2 (-0.4, -0.2)	0.92 (0.89, 0.95)	-7.8 (-10.5, -5.5)
Enterotoxigenic *Escherichia coli* (ETEC)	-0.1 (-0.2, -0.1)	0.96 (0.95, 0.98)	-3.7 (-5.3, -2.1)
Campylobacter jejuni/coli	0.0 (0.0, 0.0)	--	0.0 (0.0, 0.0)
Shigella + ETEC	-0.3 (-0.4, -0.2)	0.90 (0.88, 0.93)	-9.6 (-12.2, -7.3)
Pakistan			
Rotavirus	-0.8 (-1.0, -0.5)	0.97 (0.97, 0.98)	-2.5 (-3.2, -1.8)

Norovirus GII	-0.1 (-0.3, 0.0)	1.00 (0.99, 1.00)	-0.5 (-1.0, -0.1)
Adenovirus 40/41	0.0 (0.0, 0.0)	--	0.0 (0.0, 0.0)
Norovirus GII + Rotavirus	-0.9 (-1.3, -0.7)	0.97 (0.96, 0.98)	-3.0 (-3.9, -2.2)
Adenovirus 40/41 + Norovirus GII + Rotavirus	-0.9 (-1.3, -0.7)	0.97 (0.96, 0.98)	-3.0 (-3.9, -2.2)
Shigella	-0.8 (-1.0, -0.5)	0.98 (0.97, 0.98)	-2.4 (-3.2, -1.8)
Enterotoxigenic *Escherichia coli* (ETEC)	-0.3 (-0.5, -0.1)	0.99 (0.98, 0.99)	-1.0 (-1.5, -0.5)
Campylobacter jejuni/coli	0.0 (-0.1, 0.0)	--	-0.1 (-0.3, 0.0)
Shigella + ETEC	-1.0 (-1.4, -0.7)	0.97 (0.96, 0.98)	-3.2 (-4.2, -2.5)
Tanzania			
Rotavirus	-0.2 (-0.3, -0.1)	0.95 (0.92, 0.98)	-4.7 (-8.1, -1.8)
Norovirus GII	-0.2 (-0.3, -0.1)	0.96 (0.93, 0.98)	-4.1 (-6.9, -1.8)
Adenovirus 40/41	-0.1 (-0.2, 0.0)	0.98 (0.96, 0.99)	-2.1 (-4.3, -0.5)
Norovirus GII + Rotavirus	-0.3 (-0.5, -0.2)	0.91 (0.88, 0.95)	-8.7 (-12.4, -5.3)
Adenovirus 40/41 + Norovirus GII + Rotavirus	-0.4 (-0.6, -0.2)	0.90 (0.86, 0.93)	-10.3 (-14.4, -6.6)
Shigella	-0.1 (-0.2, 0.0)	0.98 (0.96, 1.00)	-2.0 (-4.3, -0.4)
Enterotoxigenic *Escherichia coli* (ETEC)	-0.2 (-0.3, -0.1)	0.95 (0.92, 0.97)	-5.1 (-8.1, -2.6)
Campylobacter jejuni/coli	0.0 (0.0, 0.0)	--	0.0 (0.0, 0.0)
Shigella + ETEC	-0.2 (-0.4, -0.1)	0.94 (0.91, 0.97)	-6.1 (-9.2, -3.4)

Viral vaccines administered at 2 and 4 months. Bacterial vaccines administered at 6 and 9 months. Data also reported in Figure S5.

Figures S5. Sensitivity Analysis: Assuming country-specific diarrhea incidence rates for 2021 based on the Global Burden of Disease Study, absolute (incidence rate differences) in antibiotic courses for diarrhea episodes of any etiology (any drug class) for nine enteric pathogen vaccine scenarios compared to the no vaccine scenario with 60% full vaccine efficacies against severe etiology-specific diarrhea at five sites in the MAL-ED birth cohort study.

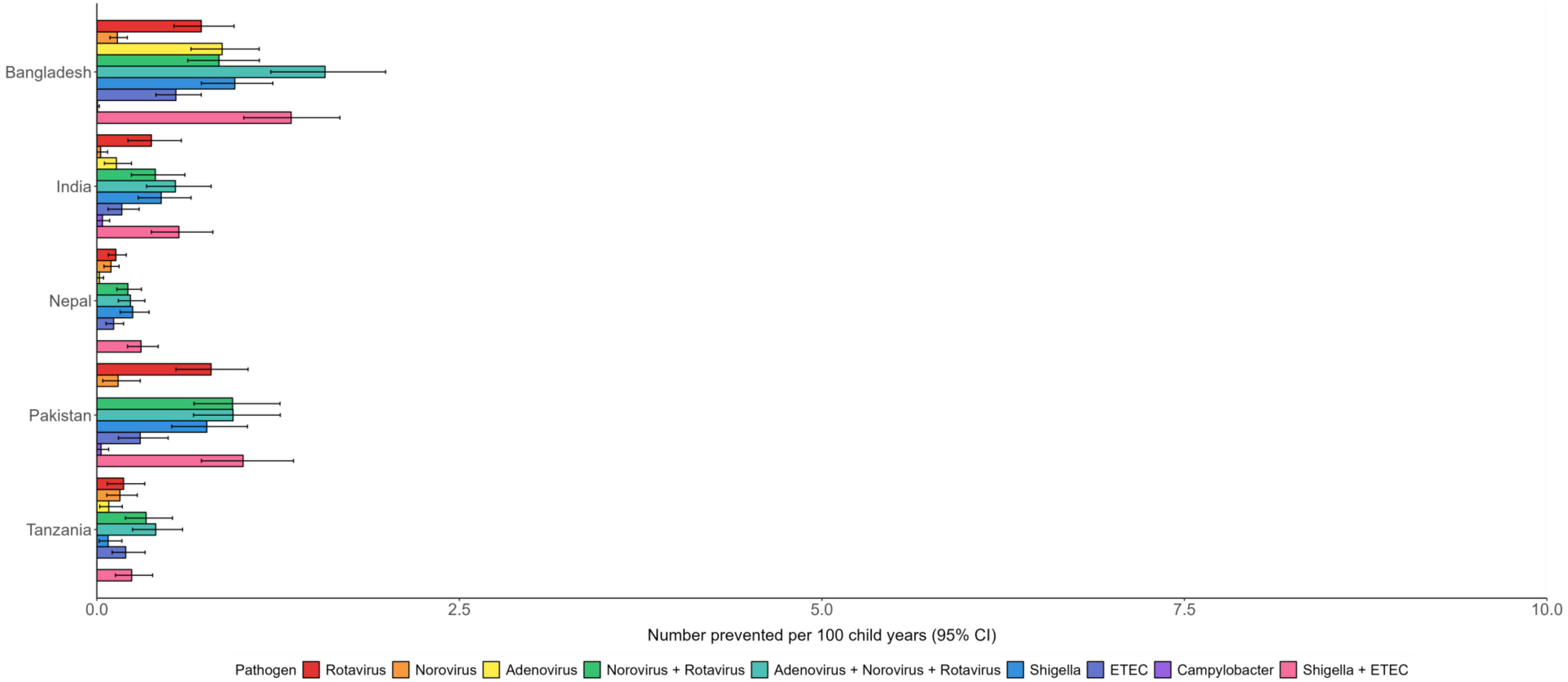

Table S7. Incidence rates of etiologic specific diarrhea (N=7458 stools) based on AFe>0.5 and Ct<35 at five sites in the MAL-ED birth cohort study.

Pathogen	AFe > 0.5		Ct < 35	
	Cases	Incidence (95% CI) (cases per 100 child years)	Cases	Incidence (95% CI) (cases per 100 child years)
Rotavirus	486	29.9 (27.6, 32.2)	765	47.1 (44.2, 49.9)
Norovirus GII	159	9.8 (8.5, 11.1)	888	54.7 (51.6, 57.7)
Adenovirus 40/41	350	21.5 (19.6, 23.5)	1185	72.9 (69.4, 76.5)
Shigella	570	35.1 (32.6, 37.5)	893	55.0 (51.9, 58.0)
Enterotoxigenic *E· coli* (ETEC)	387	23.8 (21.8, 25.8)	1779	109.5 (105.2, 113.8)
Campylobacter	37	2.3 (1.7, 2.9)	1123	69.1 (65.7, 72.6)

Table S8. Sensitivity Analysis: Absolute (incidence rate differences) and relative (incidence rate ratios) differences and percent reductions in antibiotic use outcomes (any drug class) for nine enteric pathogen vaccine scenarios compared to the no vaccine scenario with 60% full vaccine efficacies at five sites in the MAL-ED birth cohort study when diarrhea etiology was defined by the detection of a pathogen by qPCR at any quantity (quantification cycle threshold < 35).

Vaccine scenario and efficacy outcome	Incidence rate difference (cases per 100 child years)	Incidence rate ratio	Percent reduction
Rotavirus			
Antibiotic courses for bacterial diarrhea episodes	-7·5 (-8·7, -6·4)	0·93 (0·92, 0·94)	-6·9 (-7·9, -6·0)
Antibiotic courses for viral/parasitic diarrhea episodes	-9·4 (-10·6, -8·2)	0·91 (0·90, 0·92)	-8·5 (-9·6, -7·6)
Antibiotic courses for diarrhea episodes of any etiology	-9·4 (-10·6, -8·2)	0·93 (0·92, 0·94)	-7·0 (-7·8, -6·2)
Antibiotic courses overall	-9·4 (-10·6, -8·2)	0·99 (0·99, 0·99)	-1·2 (-1·3, -1·0)
Antibiotic exposures to bystander pathogens overall	-7·1 (-8·7, -5·8)	0·99 (0·99, 1·00)	-0·6 (-0·7, -0·5)
Norovirus GII			
Antibiotic courses for bacterial diarrhea episodes	-9·7 (-11·0, -8·4)	0·91 (0·90, 0·92)	-8·8 (-9·8, -7·8)
Antibiotic courses for viral/parasitic diarrhea episodes	-11·2 (-12·6, -9·8)	0·90 (0·89, 0·91)	-10·1 (-11·1, -9·1)
Antibiotic courses for diarrhea episodes of any etiology	-11·2 (-12·6, -9·8)	0·92 (0·91, 0·93)	-8·3 (-9·1, -7·4)
Antibiotic courses overall	-11·2 (-12·6, -9·8)	0·99 (0·98, 0·99)	-1·4 (-1·6, -1·2)
Antibiotic exposures to bystander pathogens overall	-7·4 (-8·9, -6·1)	0·99 (0·99, 0·99)	-0·6 (-0·8, -0·5)
Adenovirus 40/41			
Antibiotic courses for bacterial diarrhea episodes	-13·2 (-15·0, -11·5)	0·88 (0·87, 0·89)	-12·1 (-13·4, -10·7)
Antibiotic courses for viral/parasitic diarrhea episodes	-14·8 (-16·8, -13·0)	0·87 (0·85, 0·88)	-13·5 (-14·8, -12·1)
Antibiotic courses for diarrhea episodes of any etiology	-14·8 (-16·8, -13·0)	0·89 (0·88, 0·90)	-11·0 (-12·1, -9·9)
Antibiotic courses overall	-14·8 (-16·8, -13·0)	0·98 (0·98, 0·98)	-1·9 (-2·1, -1·6)
Antibiotic exposures to bystander pathogens overall	-12·0 (-14·0, -10·3)	0·99 (0·99, 0·99)	-1·0 (-1·2, -0·9)
Norovirus GII + Rotavirus			
Antibiotic courses for bacterial diarrhea episodes	-15·7 (-17·5, -14)	0·86 (0·84, 0·87)	-14·4 (-15·7, -13·3)
Antibiotic courses for viral/parasitic diarrhea episodes	-19·0 (-20·9, -17·2)	0·83 (0·82, 0·84)	-17·2 (-18·4, -16)
Antibiotic courses for diarrhea episodes of any etiology	-19·0 (-20·9, -17·2)	0·86 (0·85, 0·87)	-14·1 (-15·1, -13·1)
Antibiotic courses overall	-19·0 (-20·9, -17·2)	0·98 (0·97, 0·98)	-2·4 (-2·6, -2·2)
Antibiotic exposures to bystander pathogens overall	-13·4 (-15·4, -11·5)	0·99 (0·99, 0·99)	-1·2 (-1·3, -1·0)
Adenovirus 40/41 + Norovirus GII + Rotavirus			
Antibiotic courses for bacterial diarrhea episodes	-23·8 (-26·4, -21·5)	0·78 (0·77, 0·79)	-21·9 (-23·3, -20·5)
Antibiotic courses for viral/parasitic diarrhea episodes	-28·0 (-30·5, -25·5)	0·75 (0·73, 0·76)	-25·4 (-26·6, -23·9)
Antibiotic courses for diarrhea episodes of any etiology	-28·0 (-30·5, -25·5)	0·79 (0·78, 0·81)	-20·7 (-21·9, -19·5)
Antibiotic courses overall	-28·0 (-30·5, -25·5)	0·96 (0·96, 0·97)	-3·5 (-3·8, -3·2)
Antibiotic exposures to bystander pathogens overall	-21·0 (-23·6, -18·4)	0·98 (0·98, 0·98)	-1·8 (-2·0, -1·6)
Shigella			
Antibiotic courses for bacterial diarrhea episodes	-12·0 (-13·4, -10·6)	0·89 (0·88, 0·90)	-11·0 (-12·0, -10·0)

Antibiotic courses for viral/parasitic diarrhea episodes	-10·4 (-11·7, -9·1)	0·91 (0·90, 0·92)	-9·4 (-10·4, -8·4)
Antibiotic courses for diarrhea episodes of any etiology	-12·0 (-13·4, -10·6)	0·91 (0·90, 0·92)	-8·8 (-9·8, -8·0)
Antibiotic courses overall	-12·0 (-13·4, -10·6)	0·98 (0·98, 0·99)	-1·5 (-1·7, -1·3)
Antibiotic exposures to bystander pathogens overall	-8·7 (-10·2, -7·4)	0·99 (0·99, 0·99)	-0·8 (-0·9, -0·6)
Enterotoxigenic *Escherichia coli* (ETEC)			
Antibiotic courses for bacterial diarrhea episodes	-18·8 (-20·8, -16·9)	0·83 (0·82, 0·84)	-17·2 (-18·4, -16·0)
Antibiotic courses for viral/parasitic diarrhea episodes	-16·9 (-18·7, -15·2)	0·85 (0·84, 0·86)	-15·3 (-16·4, -14·2)
Antibiotic courses for diarrhea episodes of any etiology	-18·8 (-20·8, -16·9)	0·86 (0·85, 0·87)	-13·9 (-15·0, -12·9)
Antibiotic courses overall	-18·8 (-20·8, -16·9)	0·98 (0·97, 0·98)	-2·4 (-2·6, -2·1)
Antibiotic exposures to bystander pathogens overall	-13·7 (-15·8, -11·8)	0·99 (0·99, 0·99)	-1·2 (-1·4, -1·0)
Campylobacter jejuni/coli			
Antibiotic courses for bacterial diarrhea episodes	-11·5 (-13·0, -10·0)	0·89 (0·88, 0·90)	-10·5 (-11·6, -9·5)
Antibiotic courses for viral/parasitic diarrhea episodes	-10·5 (-12·0, -9·0)	0·91 (0·89, 0·92)	-9·5 (-10·6, -8·4)
Antibiotic courses for diarrhea episodes of any etiology	-11·5 (-13·0, -10·0)	0·91 (0·90, 0·92)	-8·5 (-9·5, -7·6)
Antibiotic courses overall	-11·5 (-13·0, -10·0)	0·99 (0·98, 0·99)	-1·4 (-1·6, -1·3)
Antibiotic exposures to bystander pathogens overall	-6·6 (-7·9, -5·4)	0·99 (0·99, 1·00)	-0·6 (-0·7, -0·5)
Shigella + ETEC			
Antibiotic courses for bacterial diarrhea episodes	-23·6 (-25·8, -21·6)	0·78 (0·77, 0·80)	-21·7 (-22·9, -20·5)
Antibiotic courses for viral/parasitic diarrhea episodes	-20·9 (-22·9, -18·9)	0·81 (0·80, 0·82)	-18·9 (-20·1, -17·7)
Antibiotic courses for diarrhea episodes of any etiology	-23·6 (-25·8, -21·6)	0·82 (0·81, 0·84)	-17·5 (-18·6, -16·4)
Antibiotic courses overall	-23·7 (-25·9, -21·6)	0·97 (0·97, 0·97)	-3·0 (-3·3, -2·7)
Antibiotic exposures to bystander pathogens overall	-18·1 (-20·6, -15·8)	0·98 (0·98, 0·99)	-1·6 (-1·8, -1·4)

Viral vaccines administered at 2 and 4 months. Bacterial vaccines administered at 6 and 9 months.

Table S9. Sensitivity Analysis: Absolute (incidence rate differences) and relative (incidence rate ratios) differences and percent reductions in antibiotic use outcomes (any drug class) for nine enteric pathogen vaccine scenarios compared to the no vaccine scenario with 60% full vaccine efficacies against severe etiology-specific diarrhea at five sites in the MAL-ED birth cohort study with 60% vaccine coverage.

Vaccine scenario and efficacy outcome	Incidence rate difference (cases per 100 child years)	Incidence rate ratio	Percent reduction
Rotavirus			
Antibiotic courses for bacterial diarrhea episodes	-0.6 (-0.8, -0.4)	0.98 (0.98, 0.99)	-1.8 (-2.4, -1.2)
Antibiotic courses for viral/parasitic diarrhea episodes	-4.0 (-4.6, -3.4)	0.92 (0.9, 0.93)	-8.5 (-9.5, -7.5)
Antibiotic courses for diarrhea episodes of any etiology	-4.0 (-4.6, -3.4)	0.97 (0.97, 0.97)	-2.9 (-3.4, -2.6)
Antibiotic courses overall	-4.0 (-4.6, -3.4)	1.00 (0.99, 1.00)	-0.5 (-0.6, -0.4)
Antibiotic exposures to bystander pathogens overall	-7.2 (-8.6, -5.9)	0.99 (0.99, 1.00)	-0.6 (-0.7, -0.5)
Norovirus GII			
Antibiotic courses for bacterial diarrhea episodes	-0.3 (-0.5, -0.2)	0.99 (0.99, 0.99)	-0.9 (-1.4, -0.5)
Antibiotic courses for viral/parasitic diarrhea episodes	-1.0 (-1.3, -0.8)	0.98 (0.97, 0.98)	-2.2 (-2.8, -1.7)
Antibiotic courses for diarrhea episodes of any etiology	-1.0 (-1.3, -0.8)	0.99 (0.99, 0.99)	-0.8 (-1.0, -0.6)
Antibiotic courses overall	-1.0 (-1.3, -0.8)	--	-0.1 (-0.2, -0.1)
Antibiotic exposures to bystander pathogens overall	-1.9 (-2.5, -1.3)	--	-0.1 (-0.2, -0.1)
Adenovirus 40/41			
Antibiotic courses for bacterial diarrhea episodes	-1.3 (-1.6, -1.0)	0.96 (0.95, 0.97)	-3.8 (-4.7, -3.0)
Antibiotic courses for viral/parasitic diarrhea episodes	-2.9 (-3.5, -2.4)	0.94 (0.93, 0.95)	-6.2 (-7.2, -5.2)
Antibiotic courses for diarrhea episodes of any etiology	-2.9 (-3.5, -2.4)	0.98 (0.97, 0.98)	-2.1 (-2.5, -1.8)
Antibiotic courses overall	-2.9 (-3.5, -2.4)	--	-0.4 (-0.4, -0.3)
Antibiotic exposures to bystander pathogens overall	-6.0 (-7.3, -4.8)	1.00 (0.99, 1.00)	-0.5 (-0.6, -0.4)
Norovirus GII + Rotavirus			
Antibiotic courses for bacterial diarrhea episodes	-0.9 (-1.1, -0.6)	0.97 (0.97, 0.98)	-2.6 (-3.3, -1.8)
Antibiotic courses for viral/parasitic diarrhea episodes	-4.9 (-5.6, -4.2)	0.90 (0.88, 0.91)	-10.4 (-11.7, -9.3)
Antibiotic courses for diarrhea episodes of any etiology	-4.9 (-5.6, -4.2)	0.96 (0.96, 0.97)	-3.6 (-4.1, -3.2)
Antibiotic courses overall	-4.9 (-5.6, -4.2)	0.99 (0.99, 0.99)	-0.6 (-0.7, -0.5)
Antibiotic exposures to bystander pathogens overall	-8.8 (-10.4, -7.4)	0.99 (0.99, 0.99)	-0.7 (-0.8, -0.6)
Adenovirus 40/41 + Norovirus GII + Rotavirus			
Antibiotic courses for bacterial diarrhea episodes	-2.0 (-2.4, -1.6)	0.94 (0.93, 0.95)	-6.0 (-7.1, -5.0)
Antibiotic courses for viral/parasitic diarrhea episodes	-7.3 (-8.2, -6.5)	0.84 (0.83, 0.85)	-15.7 (-16.8, -14.5)
Antibiotic courses for diarrhea episodes of any etiology	-7.3 (-8.2, -6.5)	0.95 (0.94, 0.95)	-5.4 (-6.0, -4.9)
Antibiotic courses overall	-7.3 (-8.2, -6.5)	0.99 (0.99, 0.99)	-0.9 (-1.0, -0.8)
Antibiotic exposures to bystander pathogens overall	-13.9 (-15.8, -12)	0.99 (0.99, 0.99)	-1.1 (-1.2, -0.9)
Shigella			

Antibiotic courses for bacterial diarrhea episodes	-4.9 (-5.6, -4.3)	0.85 (0.84, 0.87)	-14.7 (-16.0, -13.5)
Antibiotic courses for viral/parasitic diarrhea episodes	-1.8 (-2.2, -1.5)	0.96 (0.95, 0.97)	-3.9 (-4.7, -3.2)
Antibiotic courses for diarrhea episodes of any etiology	-4.9 (-5.6, -4.3)	0.96 (0.96, 0.97)	-3.7 (-4.1, -3.2)
Antibiotic courses overall	-4.9 (-5.6, -4.3)	0.99 (0.99, 0.99)	-0.6 (-0.7, -0.5)
Antibiotic exposures to bystander pathogens overall	-9.5 (-11.1, -8.2)	0.99 (0.99, 0.99)	-0.7 (-0.9, -0.6)
Enterotoxigenic *Escherichia coli* (ETEC)			
Antibiotic courses for bacterial diarrhea episodes	-2.6 (-3.1, -2.2)	0.92 (0.91, 0.93)	-7.9 (-9.0, -6.9)
Antibiotic courses for viral/parasitic diarrhea episodes	-1.1 (-1.3, -0.8)	0.98 (0.97, 0.98)	-2.3 (-2.8, -1.8)
Antibiotic courses for diarrhea episodes of any etiology	-2.6 (-3.1, -2.2)	0.98 (0.98, 0.98)	-2.0 (-2.3, -1.7)
Antibiotic courses overall	-2.6 (-3.1, -2.2)	--	-0.3 (-0.4, -0.3)
Antibiotic exposures to bystander pathogens overall	-4.6 (-5.6, -3.8)	--	-0.4 (-0.4, -0.3)
Campylobacter jejuni/coli			
Antibiotic courses for bacterial diarrhea episodes	-0.1 (-0.2, 0.0)	1.00 (0.99, 1.00)	-0.3 (-0.5, -0.1)
Antibiotic courses for viral/parasitic diarrhea episodes	0.0 (0.0, 0.0)	--	0.0 (-0.1, 0.0)
Antibiotic courses for diarrhea episodes of any etiology	-0.1 (-0.2, 0.0)	--	-0.1 (-0.1, 0.0)
Antibiotic courses overall	-0.1 (-0.2, 0.0)	--	0.0 (0.0, 0.0)
Antibiotic exposures to bystander pathogens overall	-0.2 (-0.4, 0.0)	--	0.0 (0.0, 0.0)
Shigella + ETEC			
Antibiotic courses for bacterial diarrhea episodes	-6.8 (-7.6, -6.0)	0.80 (0.79, 0.81)	-20.2 (-21.4, -19.1)
Antibiotic courses for viral/parasitic diarrhea episodes	-2.7 (-3.1, -2.2)	0.94 (0.93, 0.95)	-5.7 (-6.6, -4.8)
Antibiotic courses for diarrhea episodes of any etiology	-6.8 (-7.6, -6.0)	0.95 (0.94, 0.96)	-5.0 (-5.5, -4.5)
Antibiotic courses overall	-6.8 (-7.6, -6.0)	0.99 (0.99, 0.99)	-0.9 (-1.0, -0.8)
Antibiotic exposures to bystander pathogens overall	-12.7 (-14.7, -11.0)	0.99 (0.99, 0.99)	-1.0 (-1.1, -0.9)

Viral vaccines administered at 2 and 4 months. Bacterial vaccines administered at 6 and 9 months. Data also reported in Figures S6 and S7.

Figure S6. Sensitivity Analysis: Absolute (incidence rate) differences in antibiotic use outcomes for any drug class for nine enteric pathogen vaccine scenarios compared to the no vaccine scenario with 60% full vaccine efficacies against severe etiology-specific diarrhoea at five sites in the MAL-ED birth cohort study with 60% vaccine coverage.

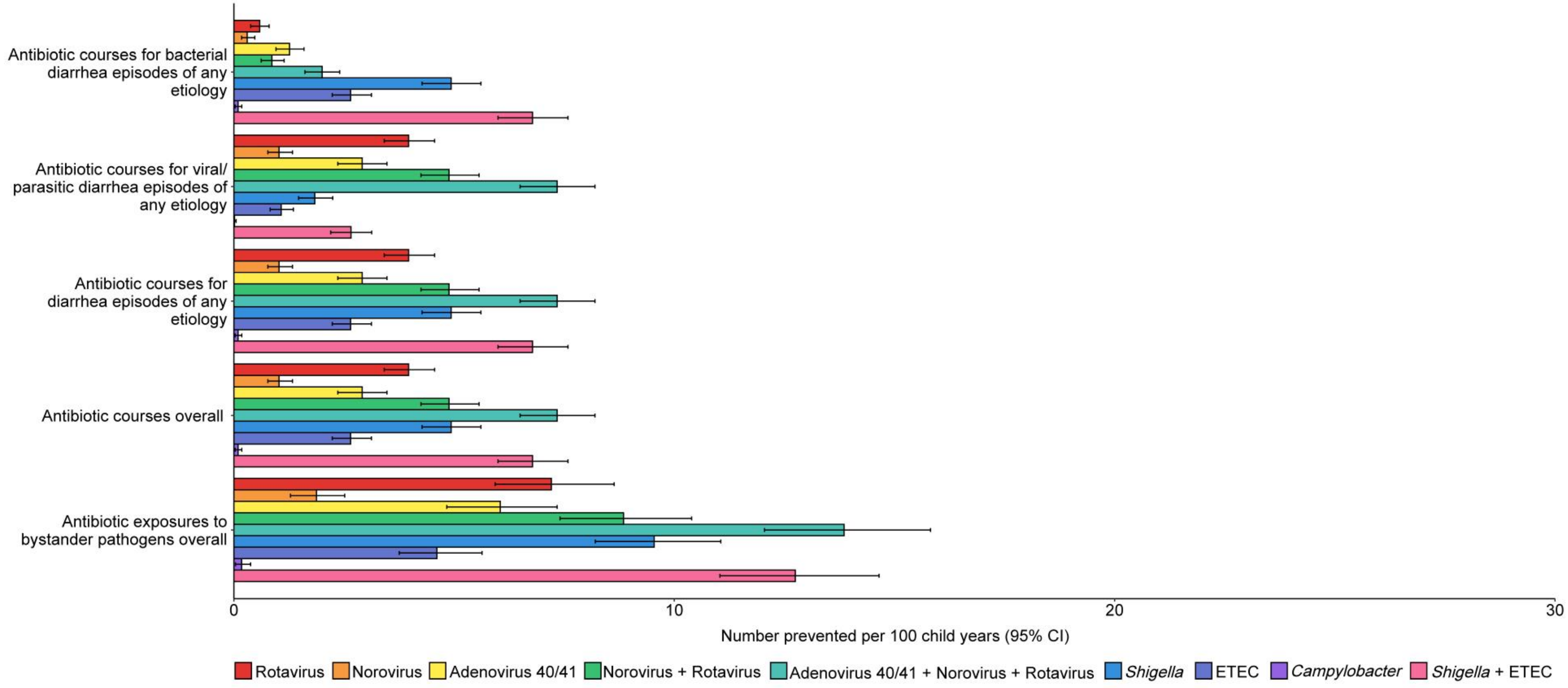

Figure S7. Sensitivity Analysis: Percent reductions in antibiotic use of any drug class for nine enteric pathogen vaccine scenarios compared to the no vaccine scenario with 60% full vaccine efficacies against severe etiology-specific diarrhoea at five sites in the MAL-ED birth cohort study with 60% vaccine coverage.

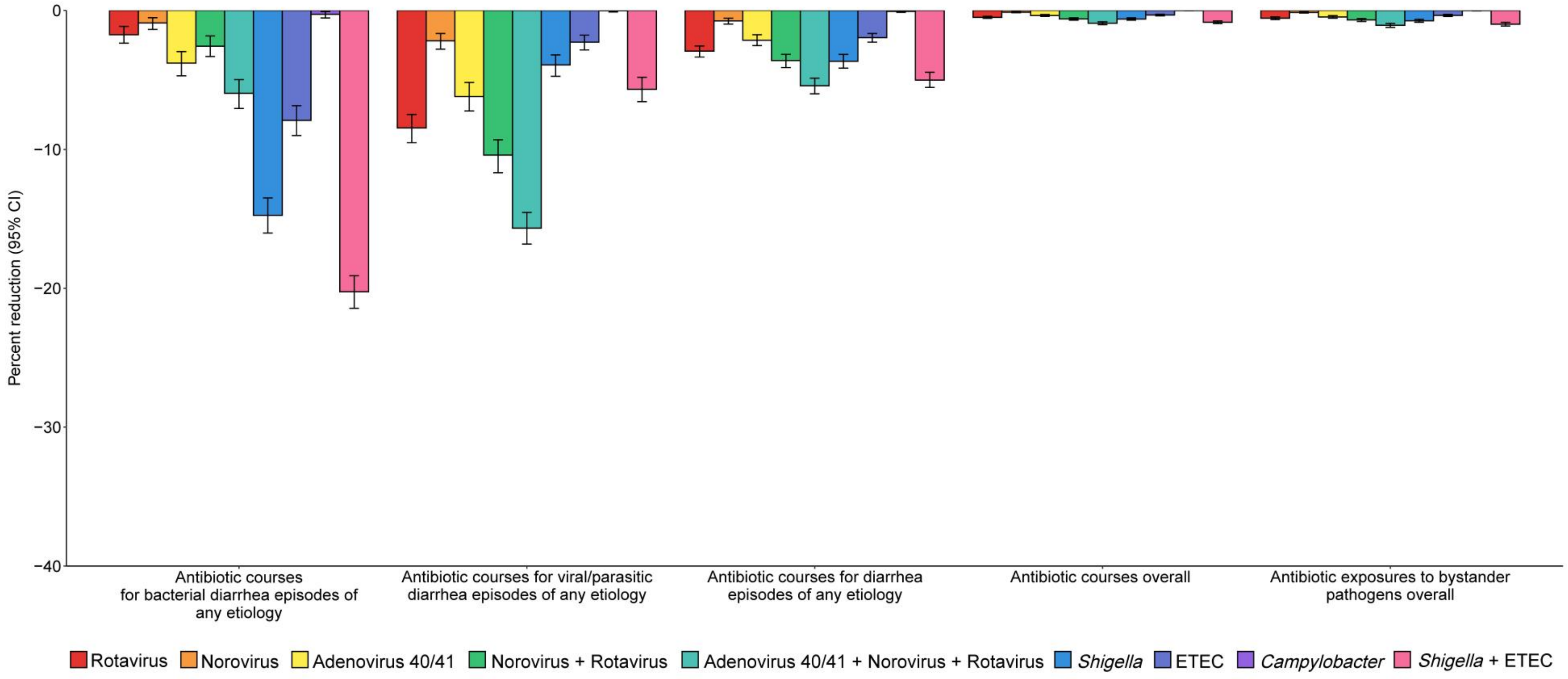

Supplement: Supplementary appendix [file mmc1.pdf]
